# Modulation of pancreatic cancer cell sensitivity to FOLFIRINOX through microRNA-mediated regulation of DNA damage

**DOI:** 10.1038/s41467-021-27099-6

**Published:** 2021-11-18

**Authors:** Pietro Carotenuto, Francesco Amato, Andrea Lampis, Colin Rae, Somaieh Hedayat, Maria C. Previdi, Domenico Zito, Maya Raj, Vincenza Guzzardo, Francesco Sclafani, Andrea Lanese, Claudia Parisi, Caterina Vicentini, Ian Said-Huntingford, Jens C. Hahne, Albert Hallsworth, Vladimir Kirkin, Kate Young, Ruwaida Begum, Andrew Wotherspoon, Kyriakos Kouvelakis, Sergio Xavier Azevedo, Vasiliki Michalarea, Rosie Upstill-Goddard, Sheela Rao, David Watkins, Naureen Starling, Anguraj Sadanandam, David K. Chang, Andrew V. Biankin, Nigel B. Jamieson, Aldo Scarpa, David Cunningham, Ian Chau, Paul Workman, Matteo Fassan, Nicola Valeri, Chiara Braconi

**Affiliations:** 1 Division of Cancer Therapeutics, The Institute of Cancer Research, London, UK; 2 TIGEM – Telethon Institute of Genetics and Medicine, Naples, Italy; 3 Institute of Cancer Sciences, University of Glasgow, Glasgow, UK; 4 Division of Molecular Pathology, The Institute of Cancer Research, London, UK; 5 Department of Medicine, University of Padua, Padua, Italy; 6 The Royal Marsden NHS Trust, London and Surrey, London, UK; 7 ARC-Net Research Centre and Department of Diagnostics and Public Health, Section of Pathology, , University of Verona, Verona, Italy; 8 West of Scotland Pancreatic Unit, Glasgow Royal Infirmary, Glasgow, UK; 9 South Western Sydney Clinical School, Faculty of Medicine, University of NSW, Sydney, NSW Australia; 10 Veneto Institute of Oncology (IOV-IRCCS), Padua, Italy; 11 Beatson West of Scotland Cancer Centre, Glasgow, UK

**Keywords:** Pancreatic cancer, miRNAs

## Abstract

FOLFIRINOX, a combination of chemotherapy drugs (Fluorouracil, Oxaliplatin, Irinotecan -FOI), provides the best clinical benefit in pancreatic ductal adenocarcinoma (PDAC) patients. In this study we explore the role of miRNAs (MIR) as modulators of chemosensitivity to identify potential biomarkers of response. We find that 41 and 84 microRNA inhibitors enhance the sensitivity of Capan1 and MiaPaCa2 PDAC cells respectively. These include a MIR1307-inhibitor that we validate in further PDAC cell lines. Chemotherapy-induced apoptosis and DNA damage accumulation are higher in MIR1307 knock-out (MIR1307KO) versus control PDAC cells, while re-expression of MIR1307 in MIR1307KO cells rescues these effects. We identify binding of MIR1307 to CLIC5 mRNA through covalent ligation of endogenous Argonaute-bound RNAs cross-linking immunoprecipitation assay. We validate these findings in an in vivo model with MIR1307 disruption. In a pilot cohort of PDAC patients undergoing FOLFIRONX chemotherapy, circulating MIR1307 correlates with clinical outcome.

## Abstract (web summary)

Understanding which patients will respond to FOLFIRINOX therapy is important for clinical outcome. Here, the authors show that the MIR1307 is increased pancreatic cancer cell lines and inhibition of the microRNA sensitises cells to treatment.’ stratifying patients to achieve the best clinical outcome. Here, the authors show that the MIR1307 is increased in a subgroup of human pancreatic cancers and inhibition of the microRNA in in vitro and in vivo models of pancreatic cancer sensitises cells to treatment.

## Introduction

Pancreatic ductal adenocarcinoma (PDAC) is a deadly disease with a survival rate lower than 10% at 5 years and a median overall survival (OS) of less than 12 months in metastatic patients^1^. Systemic treatment for PDAC has been limited to gemcitabine monotherapy for many years^2^. More recently, novel combination approaches have led to an improvement in response rate (RR) and OS^3–5^. FOLFIRINOX is a three-drug regime that increases RR compared to gemcitabine (32 vs 9%) but is associated with more severe toxicity^4^. The remarkable RR achieved with FOLFIRINOX has led to the introduction of this chemotherapy in the neo-adjuvant setting^6–9^. Despite FOLFIRINOX being reserved to patients with good performance status in current clinical practice, no evidence suggests that this parameter can predict sensitivity. In addition, the assessment of performance status is often subjective and not reliable as indicated by the analysis of quality of life in patients enrolled in the PRODIGE 4/ACCORD 11 trial^10^. Thus, biomarkers able to predict the benefit from FOLFIRINOX in PDAC patients are eagerly warranted. Genetic biomarkers have shown some promise in predicting benefit from monoclonal-antibodies^11^ or tyrosine-kinase inhibitors^12^; however, they have not proven useful to predict response to conventional chemotherapy. Growing evidence suggests that the non-protein-coding portion of the genome is crucial for cell homoeostasis, carcinogenesis, and drug response^13–16^. Several classes of non-coding RNAs (ncRNAs) have been identified^17–20^; among these, microRNAs (miRNAs or MIR) are short (18−22nt) ncRNAs that act by controlling post-transcriptional regulation of mRNA. MIRs play a key role in promoting pancreatic carcinogenesis and can aid differentiation of pancreatic cancer patients from healthy controls^21–26^. In the current study, by using a high-throughput genome-wide approach exploring the functional role of MIRs in pancreatic cancer cells exposed to chemotherapy, we identify the role of MIR1307 in mediating chemoresistance to FOLFIRINOX and propose its use as a biomarker of response to this chemotherapy regimen.

## Results

### High-throughput screening with microRNA inhibitors in PDAC cell lines

**Fig. 1:**
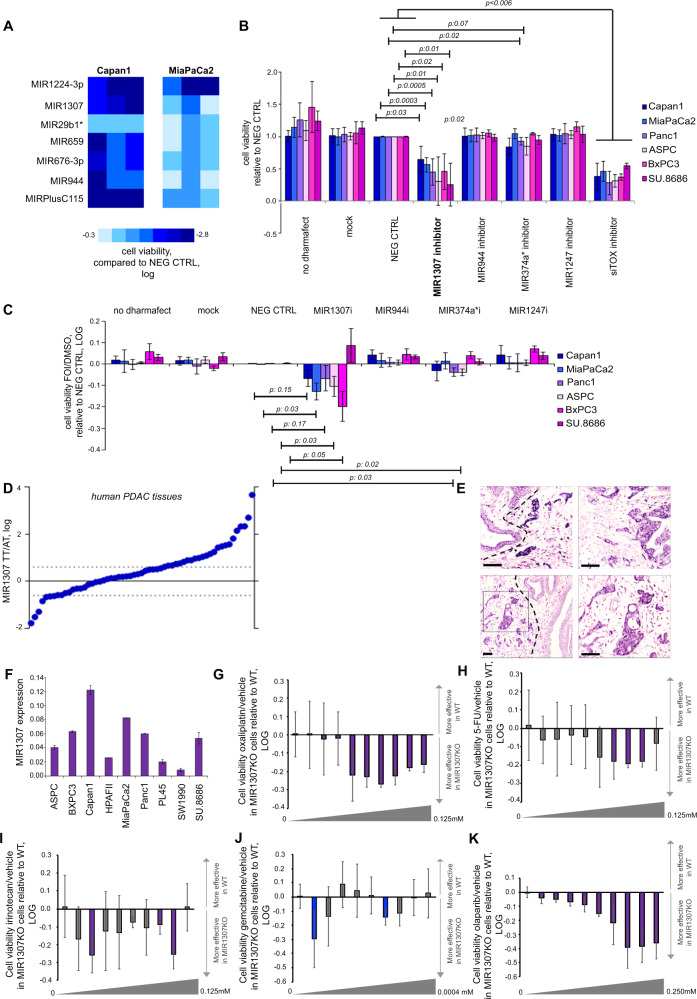
MIR1307 inhibition sensitizes PDAC cells to FOI chemotherapy in vitro and is expressed by PDAC cells in two independent cohorts of human pancreatic tissues. **A** Capan1 and MiaPaca2 cells were screened against a library of MIR inhibitors in high-throughput. Cells were reverse transfected with LNA MIR inhibitors (Exiqon) for 48 h followed by FOI treatment for 72 h before assessing cell viability by CellTiter-Blue assay. Each square indicates logarithmic value of the mean of cell viability normalized to averaged negative controls (*N* = 3), with colour code indicating the degree of change in cell viability. Only miRNA inhibitors which enhanced chemosensitivity by >30% (*p* < 0.001) in both the cell lines are shown. **B** Validation of selected HTS hits was performed in a panel of PDAC cell lines with different inhibitory probes (mirVana microRNA inhibitors, ThermoFisher Scientific) following the same protocol used for HTS. Cells were transfected with the indicated probes and then treated with FOI for 72 h. Bars indicate mean and standard deviation (SD) of five replicates. *p* values from two-sided t-test are reported. **C** PDAC cell lines were transfected with MIR inhibitors mirVana microRNA inhibitors, ThermoFisher Scientific) while they were treated with DMSO or FOI for 48 h. Bars indicated the LOG value of the ratio between FOI and DMSO and are presented normalized to NEG CTRL. Bars below the 0 line indicate that cell viability was reduced by the MIR inhibitor in the FOI treated vs the DMSO treated cells, indicating specificity for chemotherapy. Bars indicate the mean and SD of five replicates. Values from two-sided t-test are reported. **D** MIR1307 expression was assessed by Taqman assay in the tumour (TT) and adjacent tissue (AT) of 59 human PDAC samples (cohort 1). Dots represent log of the ratio between the expression in TT and that in AT for each patient. Dashed lines indicate TT > AT fold change >1.3. **E** MIR1307 was assessed by in situ hybridization in FFPE tissue from human resected PDAC. Representative pictures of four different cases of PDAC. Bars indicate 100 μm. In the bottom left quadrant, it is possible to observe positive epithelial cancer cells (black square) and negative normal ducts (right of the dashed line). **F** MIR1307 expression was assessed in human PDAC cell lines by Taqman assay. Bars represent the mean and SD of three replicates. **G** MIR1307KO and WT MiaPaca2 cells were treated with oxaliplatin (scalar concentrations as indicated in the figure) or vehicle for 48 h. Bars represent the LOG value of the ratio between drug and vehicle and are presented normalized to WT. Bars below the 0 line indicate that the reduction in cell viability was increased in MIR1307KO cells. Purple bars indicate statistically significance (*p* < 0.05). Bars indicate mean and SD of five replicates. Cell viability was reduced by 75% at the highest dose in WT. **H** 5-Fluorouracil (see above). Cell viability was reduced by 69% at the highest dose in WT. Bars indicate mean and SD of five replicates. **I** Irinotecan (see above). Cell viability was reduced by 93% at the highest dose in WT. Bars indicate mean and SD of five replicates. **J** Gemcitabine (see above). Cell viability was reduced by 60% at the highest dose in WT. Bars indicate the mean and SD of five replicates. **K** Olaparib (see above). Cell viability was reduced by 50% at the highest dose in WT.

We performed a functional genome-wide high-throughput screening (HTS) of ~1000 Locked Nucleic Acid (LNA) MIR inhibitors in human PDAC cells treated with a combination of fluorouracil (F), oxaliplatin (O), and irinotecan (I) that resembles the FOLFIRINOX regimen in vitro. Based on the Growth Inhibitory (GI)_50_ concentration of single compounds (Supplementary Fig. S1A), we selected concentrations of the three drugs that, in combination (FOI), could reproducibly reduce cell viability of PDAC cells by no more than 50% to allow identification of sensitizers (Supplementary Fig. S1B, C). HTS was performed in human PDAC Capan1 and MiaPaCa2 cell lines in triplicate. Forty-one and 84 MIR inhibitors were able to increase sensitivity to FOI by at least 30% (*p* < 0.001) in comparison to negative controls (NEG CTRLs) in Capan1 and MiaPaCa2, respectively (Supplementary Fig. S1D−G and Supplementary Dataset 1). These hits showed statistical significance where *p* value was adjusted for multiple comparisons (Supplementary Dataset 1). Amongst these, 7 MIR inhibitors were shared across the two PDAC cell lines (Fig. 1A); these included MIR1307 and MIR944 that were previously shown to modulate sensitivity to chemotherapy in other solid tumours^27,28^ and therefore were selected for further validation. As extra controls, we also added MIR1247 that was active in none of the cell lines, and MIR374a* that was active only in Capan1 cells (Supplementary Table 1). Validation experiments were carried out with different MIR inhibitory probes (mirVana probes), to exclude probe-specific effects. MIR1307 was validated to increase sensitivity to FOI in a panel of 6 human PDAC cell lines (Fig. 1B). Interestingly, the proportion of cells killed by FOI in comparison to DMSO was greater in cells transfected with MIR1307 inhibitor when compared to negative control (NEG CTRL), making the effect of this MIR more specific for chemotherapy (Fig. 1C).

### Clinical relevance of MIR1307

To investigate the clinical relevance of this candidate MIR, we assessed the expression of MIR1307 (along with MIR374a* as a control) in a series of human PDAC tissues (Supplementary Fig. 2A). Conversely to MIR374A*, MIR1307 was expressed in human pancreatic tissues and was increased in the tumour in comparison to the matched adjacent counterpart. In an extended cohort of 59 cases, MIR1307 was increased by >1.3 fold in tumour vs adjacent tissue in 29 cases (Fig. 1D). MIR1307 was strongly expressed in the tumour component of PDAC tissues when assessed by in situ hybridization (Fig. 1E and Supplementary Table 2), while areas with chronic pancreatitis adjacent to the tumour were at most weakly positive (Fig. 1E and Supplementary Fig. 2B).

### Genetic disruption of MIR1307 in PDAC cell lines affects chemosensitivity and DNA damage

**Fig. 2:**
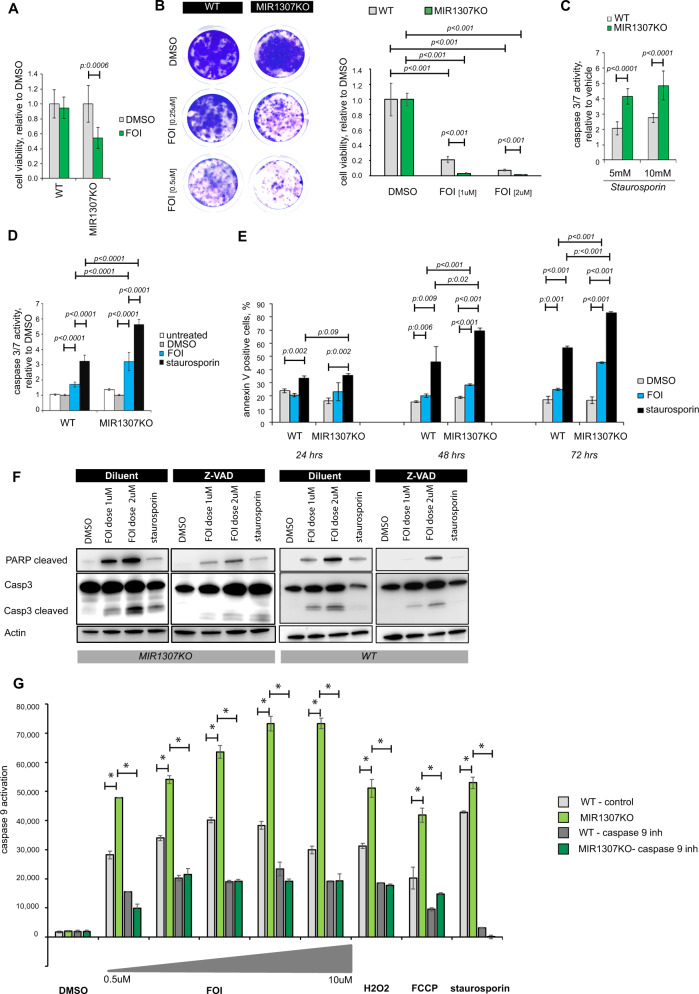
MIR1307 disruption increases apoptosis in PDAC cells. **A** MIR1307KO and WT MiaPaca2 cells were treated for 48 h with FOI or DMSO and cell viability assessed by CellTiter Blue. Values from two-sided t-test are reported. **B** MIR1307KO and WT cells were plated and treated with FOI and DMSO for 10 days before being stained with Crystal Violet. Representative pictures (left) and quantitation of four replicates with standard deviation (right) are presented. Values from two-sided t-test are reported. **C** Activation of caspase 3/7 was measured by luminescence after 24 h of treatment with staurosporin. Bars represent the mean and SD of six replicates. Values from two-sided t-test are reported. **D** Cells were treated with DMSO or FOI for 48 h before activation of caspase 3/7 activity was measured by luminescence. Staurosporin (10 μM) was added as positive control. Bars indicate the mean and SD of six replicates. Values from two-sided t-test are reported. **E** Positivity for Annexin V was measured by flow cytometry after 24, 48, and 72 h of treatment. Bars represent the mean and SD of three replicates. Values from two-sided t-test are reported. **F** Cells were treated with DMSO or FOI for 48 h in association with vehicle or Z-VAD (caspase-inhibitor) 10 μM. Source data are provided as a Source Data file. **G** Cells were plated in 96 well plates and treated with the indicated drugs for 48 h, after which caspase 9 activation was assessed by caspase 9 GLO9 assay. Increasing doses of FOI (from 0.5 to 10 μM) were used, while FFCP (10 μM), H2O2 (300 μM) and staurosporin (10 μM) were used as activators of the intrinsic apoptosis. Bars indicate the mean and SD of three replicates. * indicates *p* < 0.05 from two-sided t-test are reported.

In order to investigate the biological mechanisms involved in MIR1307-mediated resistance to FOI chemotherapy, we generated human PDAC MIR1307KO cells, by applying CRISPR-CAS9 technology in MiaPaCa2 cells that showed high baseline expression level of MIR1307 (Fig. 1F and Supplementary Fig. 3A−F). Sensitivity to FOI was increased in a dose-dependent fashion in MIR1307KO in comparison to wild-type (WT) MiaPaCa2 cell lines (Supplementary Fig. 3G, H). When the drugs were used separately, disruption of MIR1307 changed sensitivity to oxaliplatin and partially to fluorouracil (Fig. 1G, H), while no effects were recorded for irinotecan (Fig. 1I), suggesting that the activity is likely related to platinum DNA damage response. Indeed, MIR1307KO cells were confirmed to be more sensitive to olaparib, but not to gemcitabine (Fig. 1J, K). Combined FOI chemotherapy for 48 h reduced cell viability by 40% in MIR1307KO MiaPaca2 cells (Fig. 2A). Clonogenic survival assay confirmed a long-term effect of MIR1307 on chemotherapy sensitivity (Fig. 2B).

In line with these data MIR1307KO cells were more prone to undergo apoptosis than control cells when treated with staurosporin (Fig. 2C) or FOI chemotherapy as shown by increased caspase 3/7 activation and increased annexin (Fig. 2D, E). Forty-eight hours of treatment with FOI induced apoptosis with increased cleaved caspase 3 and PARP proteins (Fig. 2F), which was reversed by ZVAD caspase inhibitor. As staurosporin is acting by activating both caspase 8 and caspase 9 to finally act on caspase 3, we investigated if the FOI-induced apoptosis would be dependent on the caspase 9dependent intrinsic pathway. Indeed, caspase 9 activation was increased in MIR1307KO compared to WT cells and was abrogated by caspase 9 inhibitor (Fig. 2G).

**Fig. 3:**
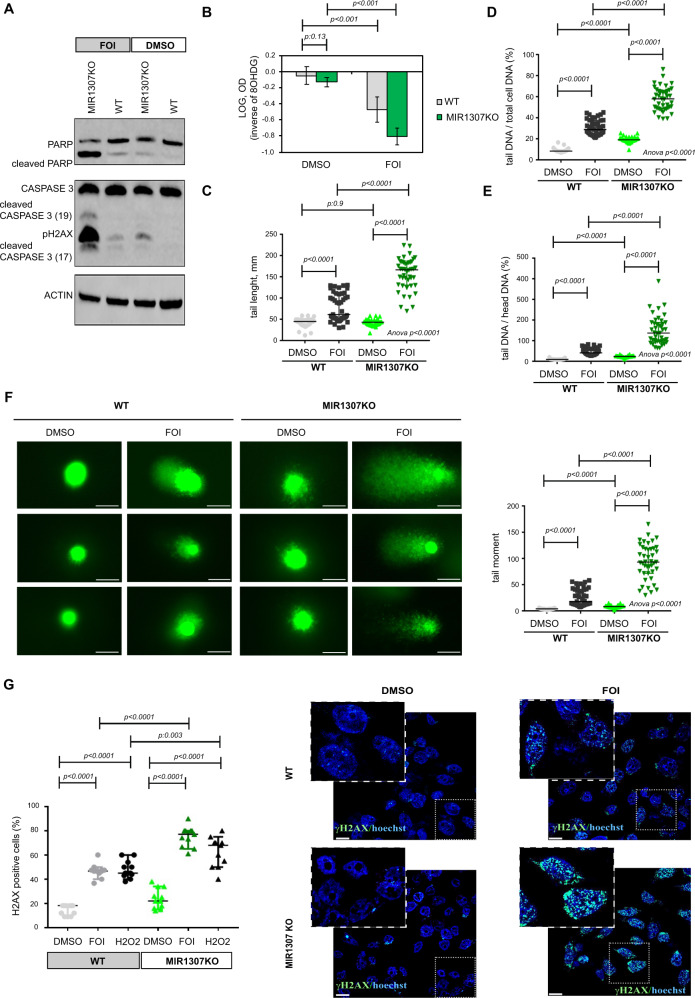
MIR1307 disruption sensitizes PDAC cells to accumulation of DNA damage. **A** Cells were treated with FOI or DMSO for 48 h and collected for western blotting analysis of the indicated proteins. Experiments were repeated twice with similar results. Source data are provided as a Source Data file. **B** 8OHdG (marker of DNA damage from oxidative stress) was measured after 48 h of treatment with DMSO or FOI. Bars indicate the mean and SD of six replicates. Values from two-sided t-test are reported. **C**−**F** COMET assay was performed to detect DNA strand breaks in cells treated with DMSO or FOI for 48 h. Representative pictures (bars indicate 100 μm) along with quantitative analyses of >40 cells (across three replicates) are represented. Error bars indicate median and interquartile ranges. Values from two-sided t-test are reported. **G** WT and MIR1307KO cells were treated with DMSO or FOI for 24 h and assessed for H2AX staining by immunofluorescence. H2O2 was used as a positive control as an inducer of DNA-damage. Quantitation (left) and representative pictures (right) are shown. The magnification bar indicates 50 μm. Dots indicate the mean value for field obtained (*n* = 10). Bars indicate median and 95% Confidence interval. Values from two-sided t-test are reported. Blue: Hoechst, Green: γH2AX.

**Fig. 4:**
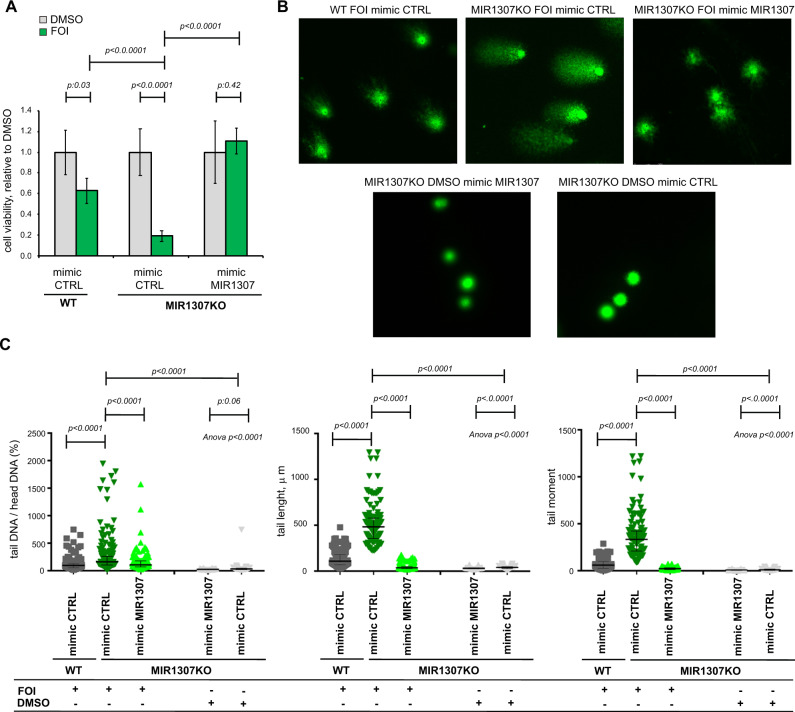
Re-expression of MIR1307 rescues chemotherapy-induced cytotoxicity and DNA damage in MIR1307KO cells. **A** Cells were treated with DMSO or FOI chemotherapy for 48 h and assessed for cell viability by CellTiter-Blue. Bars represent the mean and SD of six replicates. Values from two-sided t-test are reported. **B** Cells were treated for 48 h and COMET assay performed afterwards. Representative images are shown (magnification/scale 20×; magnification bar indicates 50 μm). **C** Quantification plots of the COMET assay are shown with median and interquartile ranges. A median of 90 cells (across three replicates) was assessed for each sample. Values from two-sided t-test are reported.

We observed significant upregulation of different markers of chemotherapy-induced DNA damage [pH2A.X (Fig. 3A), 8OHdG (Fig. 3B), DNA breaks (Fig. 3C−F)] in MIR1307KO cells treated with 48 h FOI in comparison to WT treated cells. DNA damage was confirmed by the analysis of H2AX by immunofluorescence at 24 h (Fig. 3G). Rescued expression of MIR1307 in MIR1307KO cells increased resistance to FOI chemotherapy (Fig. 4A) and protected from FOI-induced DNA damage (Fig. 4B, C).

### CLIC5 as a mediator of MIR1307 effects

**Fig. 5:**
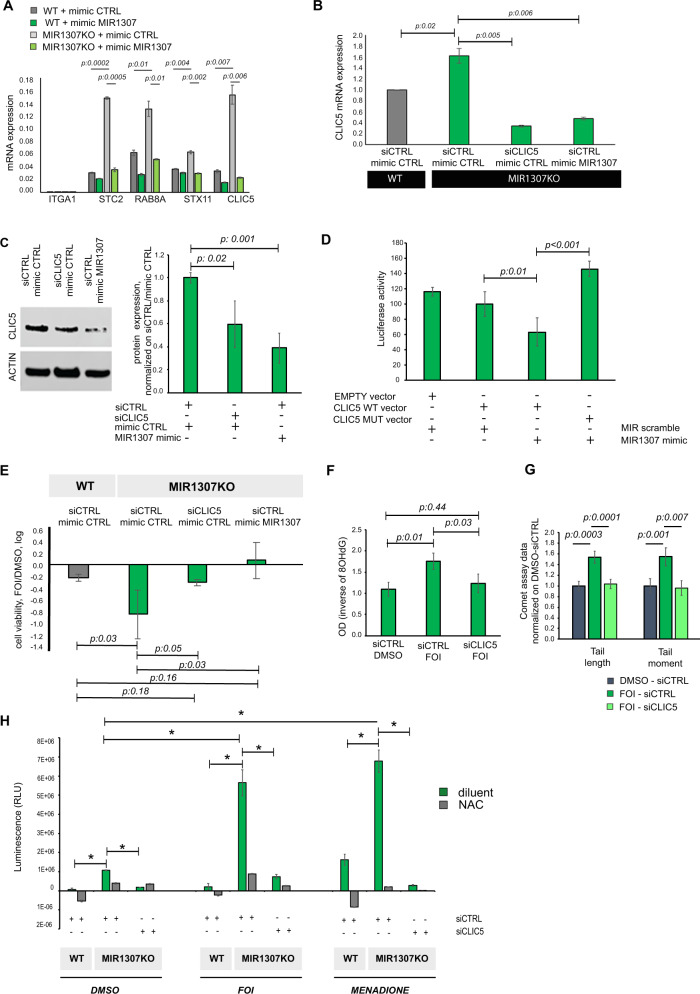
MIR1307-dependent effects are mediated by modulation of CLIC5 expression. **A** mRNA expression of indicated genes was assessed by TaqMan assay. Bars indicated the mean and SD of three replicates. Values from two-sided t-test are reported. **B** Cells were transfected for 48 h and treated with FOI for additional 48 h before mRNA expression was assessed by TaqMan assay. Bars represent mean and SD of six replicates normalized on the WT siCTRL mimicCTRL sample. Values from two-sided t-test are reported. **C** Cells were transfected for 48 h and treated with FOI for 48 h and then collected for protein assessment by western blotting. Representative pictures and quantification plots are shown here. Values from two-sided t-test are reported. Source data are provided as a Source Data file. **D** Luciferase assay was performed in MiaPaca2 cells using the vectors indicated. MIR1307 or scrambled mimic were added as indicated. Data are presented normalized to the second column from the left indicating cells transfected with CLIC5 WT vector and scrambled mimic. Bars represent the mean and SD of three independent replicates. Values from two-sided t-test are reported. **E** Cells were transfected for 48 h and treated with FOI for 48 h before assessed for cell viability by CellTiter-Blue assay. Bars represent the mean and SD of six replicates. Values from two-sided t-test are reported. Bars below the 0 line indicate reduced cell viability in FOI sample vs DMSO sample. **F** MIR1307KO cells were transfected with the indicated siRNA and DMSO or FOI chemotherapy added 24 h later. 8OHdG (marker of DNA damage from oxidative stress) was measured after 48 h of treatment. Bars indicate mean and SD of six replicates. Values from two-sided t-test are reported. As the value generated by the assay is inversely related to the DNA damage, values here are expressed as 1/actual data. **G** MIR1307KO cells were transfected with the indicated siRNA and DMSO or FOI chemotherapy added 24 h later. COMET assay was performed after 48 h of treatment. At least 200 measurements from more than five biological replicates were taken for each condition. Bars represent mean and SE. Values from two-sided t-test are reported. **H** Cells were treated with DMSO or FOI for 24 h in the presence or absence of 10 mM N-acetyl-cystein (a ROS scavenger) and ROS detected and quantitated by a luminescence-based assay. Menadione 50 μM was used as positive control (inducer of ROS). Bars represent the mean and SD of three biological replicates. * indicate *p* < 0.05 from two-sided t-test are reported.

In order to gain insights into the mechanism of action of MIR1307 we performed CLEAR-CLIP^29^ of the RNA-binding protein Argonaute to decode a genome-wide mapping of MIR:mRNA interactions present in WT cells but lost in MIR1307KO as result of MIR1307 disruption (Supplementary Figs. 4 and 5A). We mapped 5 MIR1307-mRNA chimeras in the WT cells that were not detected in MIR1307KO: these included Chloride Intracelluar Channel 5 (*CLIC5*), Integrin Subunit Alpha 1 (*ITGA1*), Ras-Related Protein Rab-8A (*RAB8A*), Stanniocalcin-2 (*STC2*) and Syntaxin 11 (*STX11*) but only *CLIC5* and *STC2* were detected at a meaningful read number (above 200 reads/million total reads) (Supplementary Table 3). Changes in mRNA expression for these genes were assessed by real time PCR (Fig. 5A) and the highest changes were confirmed to be recorded for *CLIC5* and *STC2*. CLIC5 is a member of a family of chloride channels, which form redox and pH-sensitive ion channels and have been involved in cancer progression and chemoresistance^30^. A pro-apoptotic role for mitochondrial CLICs has also been suggested^31^. Specifically, CLIC5 is found in the inner membrane of the mitochondria, where the electron exchange occurs. Deregulation of this exchange leads to generation of superoxide which damage mitochondrial DNA (mtDNA) and nuclear DNA^32^. Indeed, chemoresistance is affected by mitochondrial activity, either via direct effect from the mtDNA or via exhaustion of cells’ antioxidant capacity which activates apoptosis^32^. The CLEAR-CLIP sequencing data identified a binding site for MIR1307 in the CLIC5 coding sequence (CDS) (Supplementary Fig. 5B), which is conserved across species. In line with the hypothesis that *CLIC5* may represent a mRNA target for MIR1307, *CLIC5* mRNA expression was found to be significantly increased in MIR1307KO cells compared to WT, while its protein expression was down-regulated by the enforced expression of MIR1307 (Fig. 5B, C). MIR1307 was found to directly interact with *CLIC5* within the binding site identified by the CLEAR-CLIP as shown by luciferase assays (Fig. 5D). Expression of CLIC5 protein was inversely related to MIR1307 expression in human PDAC tissues (Supplementary Fig. 5C). Downregulation of *CLIC5* in MIR1307KO cells reproduced the functional effect on chemoresistance that was induced by enforced re-expression of MIR1307 (Fig. 5E). In addition, the effect on DNA damage accumulation by MIR1307 disruption was rescued by the inhibition of *CLIC5* in MIR1307KO cells exposed to FOI chemotherapy (Fig. 5F, G). To investigate the role of MIR1307-CLIC5 in Reactive Oxygen Species (ROS) generation we measured the levels of ROS in WT and MIR1307KO cells after transfection with siCLIC5 and siCTRL while cells were treated with and without N-acetyl-cysteine (NAC), a ROS inhibitor. We observed an increased ROS generation in MIR1307KO cells, which was remarkably enhanced after 24 h of FOI treatment. This finding was rescued by inhibition of CLIC5 in MIR1307KO cells, suggesting that MIR1307 mediates ROS generation via CLIC5 (Fig. 5H). *CLIC5* expression was found downregulated in the squamous subtype vs the progenitor subtypes (adjusted p:0.02) in the ICGC (International Cancer Genome Consortium) cohort, in line with the data on response to FOLFIRINOX in the COMPASS study (Supplementary Fig. 5D).

### Validation of the effects of MIR1307 inhibition in another PDAC cell line

**Fig. 6:**
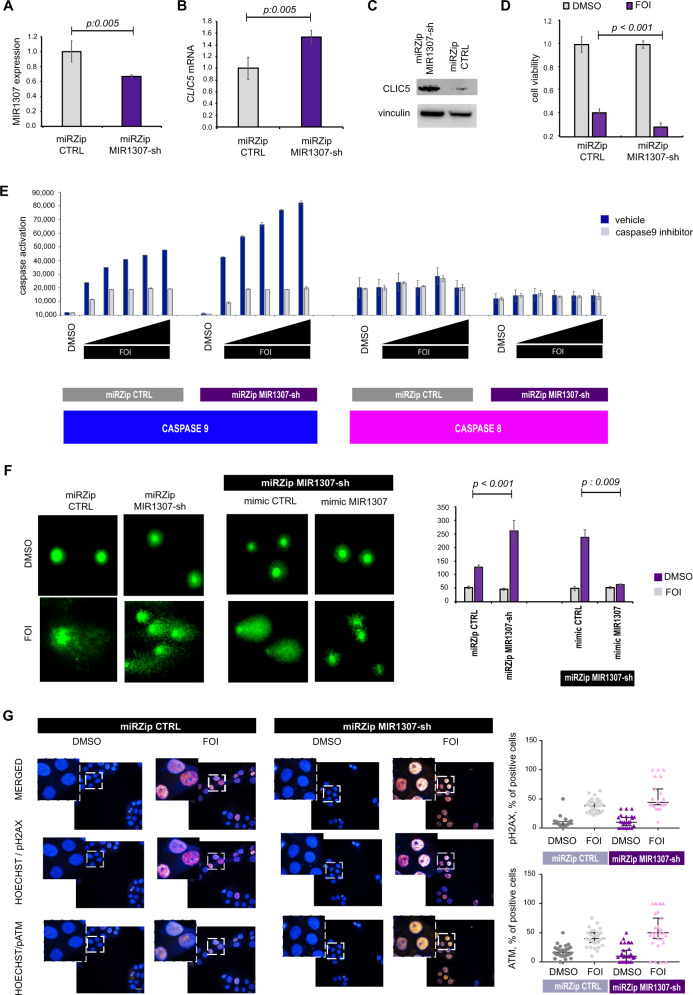
Validation of MIR1307-mediated effect in an independent in vitro PDAC model. **A** Capan 1 cells were stably infected with miRZip lentiviral vectors expressing MIR1307-sh or CTRL probes. MIR1307. **A** miRZip MIR1307-sh cells had reduced MIR1307 expression when detected by Taqman assay. Please note that it is recognized that Taqman assays can detect the MIR1307-sh produced by the vector and therefore justifies the lack of >90% MIR1307 expression. Bars represent mean of three biological replicates with SD. Values from two-sided t-test are reported. **B** CLIC5mRNA was assessed by real time PCR. Bars represent the mean and SD of three biological replicates. Values from two-sided t-test are reported. **C** CLIC5 and vinculin protein expression were assessed by western blotting. **D** Cells were plated in 96-well plates and treated with DMSO or FOI for 48 h before assessing cell viability by CellTiter Blue. Bars represent the mean and SD of 12 replicates repeated in two separate occasions. Values from two-sided t-test are reported. **E** Capan 1 cells were treated with scalar concentrations of FOI (from 0.5 to 10 μM) in presence of absence of caspase 9 inhibitor for 48 h before being assessed for caspase activation. Bars represent the mean and SD of three independent replicates. **F** Capan 1 cells were treated for 48 h and COMET assay performed afterwards. Representative images are shown (magnification/scale 20×, magnification bar indicates 50 μm). At least 50 cells (across three replicates) were assessed for each sample. Bars represent mean and standard error. Values from two-sided t-test are reported. **G** Cells were treated with DMSO or FOI for 24 h and fixed before being stained with the indicated fluorescent antibodies. Representative pictures (left) along with quantitation (right) of the percentage of positive cells on the total cells in the field, with at least five fields assessed per replicate in six biological replicates. Bars represent the mean and 95% Confidence interval. The magnification bar indicates 20 μM.

To validate our findings, we studied the functional effect of MIR1307 silencing in another PDAC cell line. Capan 1 cells were stably infected via a lentiviral vector to express an anti-MIR1307 short hairpin (miRZip MIR1307-sh) or a scrambled probe (miRZip CTRL) (Fig. 6A). CLIC5 mRNA and protein expression was increased in Capan1 miRZip MIR1307-sh compared to miRZip CTRL (Fig. 6B, C). Capan1 miRZip MIR1307-sh cells were more sensitive to FOI than miRZip CTRL cells (Fig. 6D), had increased apoptosis at 48 h (Fig. 6E), and displayed increased DNA damage as shown by the COMET assay (Fig. 6F). We observed increased staining of ATM and pH2AX at 24 h in miRZip MIR1307-sh compared to miRZip CTRL (Fig. 6G).

### In vivo validation of the role of MIR1307 in chemoresistance

**Fig. 7:**
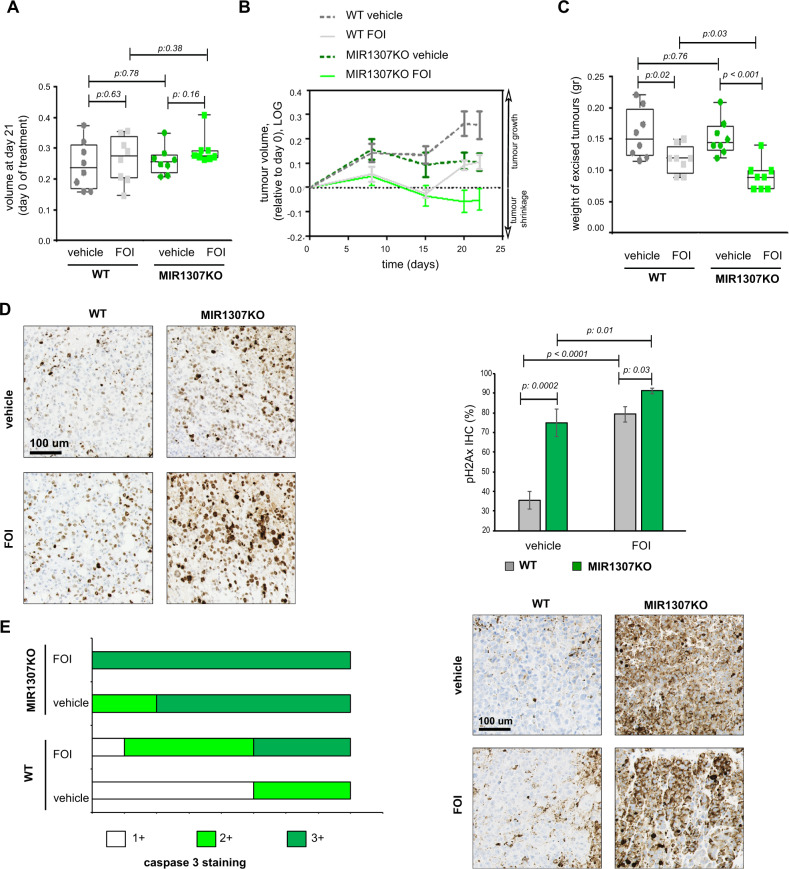
MIR1307 disruption increases chemosensitivity of PDAC xenografts. WT or MIR1307KO MiaPaca2 cells were injected subcutaneously in the flank of NSG mice (*N* = 16 each) and monitored for growth by caliper. When tumours became visible and measurable (day 21), mice were randomized to be treated with a weekly intraperitoneal vehicle (saline alone) or combination of oxaliplatin (3 mg/kg), fluorouracil (25 mg/kg), and irinotecan (25 mg/kg) for 4 weeks before being sacrificed. **A** Tumour volume at day 21 was comparable across the groups. Minimum to maximum values are presented. The box indicates the 95% confidence interval with median depicted within the box. Values from two-sided t-test are reported. **B** Tumour volume data during treatment are shown. Data are normalized to baseline pre-treatment tumour size (day 21). For statistical data please see Table S5. Two-way ANOVA test: p 0.05. **C** Excised tumours were weighed before being stored at −80 for analyses. Median values along with minimum to maximum values are shown. Values from two-sided t-test are reported. **D** Excised tumours were fixed in formalin and included in paraffin. pH2A.X was assessed by IHC and expressed as % of positive cells in each sample. Representative picture and quantification plots are shown. Values from two-sided t-test are reported. **E** Caspase 3 protein expression was assessed by IHC with a semiquantitative score. Each sample was scored as mild (1+), moderate (2+), and strong (3+) expression. Representative picture along with quantification plots are shown.

**Fig. 8:**
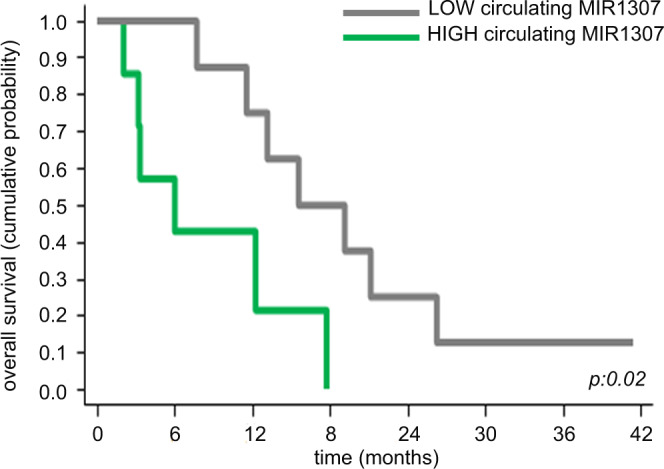
Circulating MIR1307 predicts benefit from FOLFIRINOX. Plasma was collected from advanced PDAC patients undergoing FOLFIRINOX chemotherapy and subjected to RNA extraction. MIR1307 expression was assessed by droplet digital PCR. Cohort was split according to median MIR1307 expression in low and high MIR1307. Kaplan−Meier curve for correlation with overall survival (OS) is shown.

When MiaPaca MIR1307KO and WT tumour xenografts were treated with FOI or vehicle control, we observed activity of FOI chemotherapy in both groups, but the response was more durable in the MIR1307KO compared to WT group, suggesting that the DNA damage repair (DDR) is limited in the tumours lacking MIR1307 (Fig. 7A−C and Supplementary Table 4). No statistically significant differences in tumour growth were noted between WT and MIR1307KO mice in absence of FOI treatment until 21 days post-inoculation, even though the tumour growth appeared slower in MIR1307KO. However, changes in tumour growth between MIR1307KO and WT were enhanced in the FOI-treatment arm. Although we cannot exclude an effect of MIR1307 on tumour growth, our data support an effect of MIR1307 on FOI resistance. In line with these data, we detected statistical significant tumour shrinkage only in MIR1307KO mice treated with FOI (Fig. 7B). In addition, the weight of excised tumours in FOI-treated mice was reduced by 25 and 41% in comparison to vehicle in WT and MIR1307KO groups respectively (Fig. 7C). Expression of markers of DNA damage was increased in MIR1307KO tumour xenografts compared to WT. In line with our in vitro data, MIR1307 disruption makes tumours more prone to undergo DNA damage in vivo as highlighted by the high pH2Ax (Fig. 7D) and caspase 3 (Fig. 7E) in FOI-treated MIR1307KO xenografts compared to WT. Last, we verified the potential of MIR1307 to be used as a biomarker of response to FOLFIRINOX chemotherapy in patients undergoing first line palliative treatment. Patients with unresectable PDAC treated with FOLFIRINOX were retrieved from the SSCCG (Screening Study of Genetic Changes in Colorectal, Gastrointestinal and Hepatobiliary Cancers) study, which allowed collection of clinically annotated baseline pre-treatment plasma samples (Supplementary Table 5). Analysis of circulating MIR1307 was performed in these samples by digital droplet PCR. High expression of circulating MIR1307 was associated with reduced benefit from FOLFIRINOX chemotherapy in inoperable PDAC patients (Fig. 8) and was independent from disease stage (p:0.81) and performance status (p:0.58).

## Discussion

FOLFIRINOX is a three-drugs combination regimen that is widely used in the clinical management of PDAC patients. In the adjuvant setting, FOLFIRINOX has provided a significant benefit over gemcitabine with a 20-month increase in median overall survival and a 20% reduction in tumour recurrence at 3 years^33^. In advanced unresectable PDAC, FOLFIRINOX represents the most effective chemotherapy regimen with a response rate > 30%. Preliminary data from the ESPAC5 trial also suggested a possible role for neoadjuvant FOLFIRINOX in improving survival rate of resectable PDAC patients^34^. Despite these exciting results, FOLFIRINOX is still reserved to a minority of patients due to the severe toxicity profile with up to 75% of patients experiencing grade 3 or 4 side effects^35^. These data underline the need for biomarkers that support a programme of advanced supportive care to improve tolerability in patients who derive benefit from FOLFIRINOX, while sparing unnecessary toxicities to non-responders. Despite remarkable advancement in the field of biomarker development, most of the successes have been recorded for the identification of targetable molecular alterations that predict the benefit to targeted therapies. Unfortunately, no biomarkers have yet been identified to personalize cytotoxic chemotherapy delivery in a scenario where more and more regimens are becoming available. Whole transcriptomic profiles delineated signatures of homologous recombination deficiency that are associated with platinum response in vitro in human PDAC cell lines^36^. DDR gene mutations have been associated withFOLFIRINOX response in retrospective cohorts of PDAC patients^37,38^. These analyses require appropriate tissue quantity and quality for DNA analyses.

In our study, we provide an additional liquid biopsy test that can be widely applicable and cost-effective to select FOLFIRINOX responders and deserve clinical investigation in PDAC patients. Functional biological high-throughput analyses have been applied in this work to explore the role of MIRs in the dynamics of drug response in live human PDAC cells exposed to FOLFIRINOX chemotherapy. The advantage of this approach over descriptive comparative analyses in sensitive vs resistant models relies on the possibility to unmask responsive mechanisms of chemoresistance that occur in (1) living cells under treatment stress and (2) in subclones of chemo-resistant cells. Indeed, in biliary cancers, we have previously shown that chemotherapy resistance is based on a functional response of a subclone population, whose growth and activity would be masked from descriptive analyses^15^. Our choice of investigating MIRs inhibitors as mediators of chemoresistance is based on (1) the potential of circulating MIRs to be detected in patients’ plasma through non-invasive tests^39^, (2) the greater accuracy of detecting the presence (rather than absence) of circulating MIRs in human blood samples, and (3) the association between circulating and tissue MIRs^40^. Through these studies, we identified a promising microRNA, MIR1307, which mediates chemoresistance in vitro and in vivo and reflects clinical benefit in a pilot cohort of advanced PDAC patients. From a clinical perspective, these data warrant further investigation and assessment in prospective clinical studies. Our study indicates feasibility to detect and quantitate circulating MIR1307 RNA copies, thus providing potential quantitative threshold for prospective assessment. Based on our biological data the action of MIR1307 seems to be related to platinum DNA damage. Indeed, modulation of MIR1307 expression affects mainly sensitivity to platinum-containing regimens. We observed MIR1307 to alter the expression of CLIC5. CLIC5 is known to be involved in ion transport through cellular compartments^41^. Proteins of the CLIC family are regulated by cytoskeleton filaments and modify solute transport (not only chloride) during biological processes such as apoptosis^42^. CLIC4 and CLIC5 are the only members of the family to be expressed in the mitochondrial membrane, with CLIC4 being detected in the outer membrane and CLIC5 being abundant in the inner mitochondrial membrane^30^. Mitochondria harbour a plethora of regulated ion channels whose function is related to ion/metabolite transport and to fine-tuning of mitochondrial membrane potential, as well as of reactive oxygen species release. Alterations in mitochondrial DNA were shown to result in chemoresistance and may constitute the link between deregulation of MIR1307 and drug resistance^43^. Indeed, CLIC4 was found to be reduced in cancer cells and have onco-suppressive properties^44^, and low CLIC5 expression seems to be associated with poor prognosis in breast cancer patients^30^. We observed a higher expression of CLIC5 in the classical subtype compared to the squamous subtype. Data from the COMPASS trial looking at molecular biomarkers in PDAC patients undergoing FOLFIRINOX clearly showed that response rate was significantly greater in the classical subtype compared to the basal-like (34% vs 8%). These findings support our data that the MIR1307-induced reduction of CLIC5 may be responsible for FOLFIRINOX resistance in the squamous (basal-like) subtype^45^.

Lastly, we would like to draw attention to a new methodology; in this study, we employed an adapted version of CLEAR-CLIP described by Moore et al.^29^. The use of RNA chimeras provides the advantage of identifying UV crosslinked AGO-miRNA-mRNA complexes bound in a single dataset. However, as with the traditional HITS-CLIP method, it requires the use of radioisotope labelling of RNAs at 5′ ends making its application limited to specific labs with dedicated areas. Moreover, the natural decay of radioactive reagents reduces the signal in autoradiography, making it difficult to optimize across experiments. Here we confirm that the use of fluorescent probes can retain stable signal in time and can be handled in any lab, enhancing the potential wide use of the method for inter-lab cross-comparison of results^46^.

## Methods

### Cell line

PDAC cell lines were purchased from the American Type Culture Collection. Cells were regularly tested negative for Mycoplasma and authenticated through Short Tandem Repeat (STR) analysis. All cancer cells were cultured in Dulbecco’s Modified Eagle Media with 10% fetal bovine serum (ThermoFisher Scientific, UK), at 37 °C with 5% carbon dioxide.

### Cell viability

Cell viability was measured by CellTiter-Blue^®^ Assay (Promega, Madison, WI, USA) and the GI_50_ derived using Prism Software (Graphpad, La Jolla, USA).

### High-throughput-screening (HTS)

A human LNA MIR inhibitor library (miRCURY LNA, version 3) from Exiqon (Qiagen, UK) was purchased. The library was distributed across 5 × 384-well plates (Greiner Bio-One, Frickenhausen, Germany). Each plate included two negative controls (LNA negative A, LNA negative B from Exiqon; each in quadruplicate), a positive control in quadruplicate (siTOX from Dharmacon Lafayette, Colorado, USA). Additional controls for each plate were: mock controls (quadruplicates), only medium (quadruplicate), and no cells (× 20). Transfecting solution with medium and Dharmafect (Dharmacon, Lafayette, Colorado, USA) was added to the central wells of 384-well plates; MIR inhibitors were added by dispensing 500 nL solution from a source plate containing the inhibitors at a concentration of 5 μM in PBS, into the central 320 wells of a 384-plate. Thirty μl of cell solution was then added to each well to have a final concentration of 1000 cells and 50 nM of MIR inhibitors per well. Forty-eight hours later, compounds were added by dispensing 125 nL compound solution from a source plate containing the compounds at a concentration of 4 μM (fluorouracil), 4 μM (oxaliplatin), and 2 μM (irinotecan) in 3% DMSO. Cell viability was measured 48 hours later by CellTiter-Blue® Assay (Promega, Madison, WI, USA). The cell viability measurement from each hit was normalized to that of the averaged negative controls across five plates. Each cell line was tested in triplicate. Statistical significance (*p* < 0.05) was determined by two-sided t-test across three replicates.

### Validation experiments

The same protocol used for the HTS was used for the validation experiments. However, mirVana inhibitors probes were used (Life Technologies, Paisley, UK), and cells were treated either with a combination of chemotherapy (FOI) or with 10% DMSO.

### Human tissues

The human PDAC tissues were collected under the approval of the Ethical Committee for Clinical Research at the Royal Marsden NHS Trust (Panther study: CCR 4192). All tissues were collected with informed consent. Formalin-fixed paraffin-embedded (FFPE) tissues were recovered from 60 early PDAC that underwent resection between 1999 and 2015. RNA was extracted from the tumour and the matched non-tumour component after microscopic dissection by using the Ambion RecoverAll kit (ThermoFisher Scientific, Massachusetts, USA). A second cohort from the University Hospital of Padua (ethics #0010416) was used for the in situ hybridization.

### Real-time PCR

RNA was extracted using Trizol (Invitrogen, Carlsbad, CA, USA). Reverse transcription was performed with TaqMan microRNA reverse transcription kit (Life Technologies, Paisley, UK), and MIR expression assessed by qPCR with TaqMan assay and normalized to that of RNU48 (Life Technologies, Paisley, UK).

### Western blot

Immunoblotting was performed as previously described^20^ using the LiCOR imaging system (Lincoln, Nebraska USA). The following primary antibodies from Cell Signalling Technology (London, UK) were used: PARP (#9542), cleaved PARP (#5625), Caspase 3 (#9662), cleaved Caspase 3 (#9664), pH2A.X (#9718), as well as CLIC5 (AV35262, Sigma-Aldrich, Gillingham, UK) and Beta-Actin (Clone C4, MP Biomedicals, Loughborough UK).

### Luciferase assays

Cells were transfected with 200 ng of CLIC5-pMirTarget or pMiRTarget CTRL (Origene, Rockville, MD, USA) with Promofectin Transfection Reagent (PromoCell GmbH, Heidelberg, Germany) and the luciferase activity measured after 48 h using the Dual Glo Assay system (Promega, Madison, WI, USA) according to the manufacturer’s protocol in a multiwell plate luminometer (Perkin-Elmer, Seer Green, Beaconsfield, UK). Luciferase activity was normalized to that of renilla activity for each transfected well.

### MIR1307-KO generation through CRISPR-CAS9

MiaPaca2 cells were transfected with a pair of guide RNA (gRNA) probes (pCAS-Guide-EF1a-GFP; Blue Heron Biotech, Bothell, WA, USA) using Lipofectamine 3000 reagent (ThermoFisher Scientific, Waltham Massachusetts, USA). The sequence of the gRNA probes was as follows: gRNA3 5′ CGTCGGTCGTGGTAGATAGG 3′; gRNA4 5′ AATCTCGACCGGACCTCGAC 3′). Forty-eight hrs later GFP positive cells were sorted with a BD FACSAria II (BD Biosciences, San Jose, CA, USA) and maintained in culture. Genome editing was verified at day 17 using the Indel identification kit (Clontech Laboratories, Mountain View, CA, USA). Cells were then enriched for the edited clones by performing serial dilution. The final assessment of the successful genome editing was performed by sequencing and real-time PCR.

### Clonogenic assay

Cells were seeded onto Matrigel-coated 24-well plates (200 cells/well) and treated with indicated compounds or vehicle control (Dimethyl sulfoxide, DMSO; Sigma-Merck KGaA, Darmstadt, Germany) for 10 days. After washing and fixation, the cells were stained with 0.5% Crystal Violet (Bio Basic Inc., Markham, Canada) in 25% methanol for 10 min. Cell colonies were then photographed and counted.

### Generation of stable clones through lentiviral vectors

miRZip lentivector expressing anti-miR1307-3p (miRZip MIR1307-sh) or scrambled probe (miRZip CTRL) were purchased from System Bioscience (Palo Alto, CA). Capan 1 cells were plated in a complete medium. Twenty-four hours later complete medium containing 10 μg/mL of polybrene (Sigma) was added. Lentiviral vector was added and incubated for 24 h before replacing the medium. After 72 h infected cells were selected by puromycin. The presence of GFP was confirmed by fluorescence microscopy (Zeiss AxioObserver Z1 Microscope, Cambridge UK).

### Apoptotic assays

Apoptosis was assessed by using the Caspase-Glo® 3/7, Caspase-Glo® 9, and Caspase-Glo® 8 Assay Systems (Promega, Madison, WI, USA) and Dead Cell Apoptosis Kit with Annexin V FITC (ThermoFisher Scientific, Waltham, Massachusetts, USA), following the manufacturers’ instructions. BD FACSAria II (BD Biosciences, San Jose, CA, USA) was used for flow cytometry. Cells treated with 10 μM staurosporin were used as a positive control. The fluoromethyl ketone peptide Z (LEHD-FMK) was used as caspase 9 inhibitor at a concentration of 20 μM (BD Bioscience, San Diego, CA).

### Immunofluorescence

Immunofluorescence staining was performed in a black clear 96-well plate. Cell medium was removed, and cells were fixed with a solution of PBS containing 4% PFA (ThermoFisher) for 15 min at room temperature. Cells were then washed gently with PBS before being permeabilised by a solution of PBS containing 0.2% Triton X100 (ThermoFisher) for 15 min at room temperature. Cells were gently washed with PBS and blocked with a solution of PBS containing 1% BSA for 1 h at room temperature. Blocking was followed by immunostaining with the following antibodies: mouse anti-human pATM (Biolegend, San Diego USA), mouse anti-human Alexa Fluor 647 H2Ax (ser139) (Biolegend, San Diego USA), rabbit anti-mouse Alexa Fluor 568 (Abcam, Cambrigde UK).

### ROS assay

Reactive oxygen species (ROS) generation was assessed by using ROS-Glo™ H2O2 Assay Assay Systems (Promega, Madison, WI, USA), following the manufacturers’ instructions. Menadione (50 μM) were used as positive controls. NAC (N-acetyl-cystein) 10 mM was used as ROS scavenger.

### DNA damage assays

A plate-based immunoassay was used to detect the concentration of 8-oxo-dG, a marker of oxidative DNA damage (HT 8-oxo-dG ELISA Kit, R&D Systems, Minneapolis, MN, USA), and The Comet Assay Single Cell Gel Electrophoresis Assay (CN 4250-050-K; R&D Systems, Minneapolis, MN, USA) was used to measure DNA strand breaks following manufacturers’ instructions.

### Statistical analyses

Statistical analyses were performed by GraphPad Prism 6 (La Jolla, CA, USA). Results are expressed as mean ± SD, unless indicated otherwise. Groups that were normally distributed were compared with either a two-tailed Student’s *t-*test (for analysis of two groups) or using a one-way or a two-way ANOVA to compare multiple groups. Non-parametric data were analyzed using a Wilcoxon and a Mann−Whitney *U* test when comparing two groups. Significance was accepted when *p* was less than 0.05.

### Circulating MIR

Patients with unresectable pancreatic carcinoma undergoing FOLFIRINOX chemotherapy were enroled under the research protocol CCR3085 (SSGCC) that has received approval from the Research Ethics Committee, London. The study protocol conformed to the ethical guidelines of the 1975 Declaration of Helsinki. Baseline whole blood was collected in EDTA-treated tubes; cells were removed by centrifugation for 15 min at 1,500 × *g* using a refrigerated centrifuge (4 °C). The supernatant was collected and centrifuged for further 10 min at 1,500 × *g* at 4 °C. The resulting supernatant was designated plasma and was transferred into clean RNAse free tubes and stored at −80 °C. RNA was extracted from 200 uL using the miRCURY RNA isolation kit (Exiqon, Qiagen, UK) following supplier instructions. Fixed starting volume was maintained for each sample. Digital droplet PCR (BioRad, Berkeley, CA, USA) was performed as previously described^47^ using the MIR1307 TaqMan probe (Life Technologies, Paisley UK). A no-template control and a negative control for each reverse transcription reaction were included in every assay and at least 10,000 droplets were assessed for each sample. MiR-1307 expression was assessed blinded to clinical data. In the Kaplan Meier analysis, patients were divided into two groups: low MIR1307 and high MIR1307 according to the median value.

### irCLEAR-CLIP

Cells were seeded and 24 h later treated with FOI. The following day cells were UV-crosslinked at 254 nm with Spectrolinker XL-1000 UV Crosslinker (Spectronics Corporation, NY, USA). Cells were then scraped, collected, and frozen at −80 °C for subsequent analysis. A modified version of CLEAR-CLIP as described by Moore et al.^29^ was used with small modifications and summarized in Supplementary Fig. 4. To avoid the use of radioactive tag nucleosides, the 3′ linker was modified with click chemistry conjugation as per Zarnegar et al.^45^ in order to bind an IRDye800CW probe (LI-COR Biotechnology, UK) for enabling LI-COR visualization of the transferred miRNA-RNA-AGO2 complex chimeras in nitrocellulose filters. Specific barcodes were then designed in order to pool samples for next-generation sequencing with MiSeq (3′ linker and Index barcode sequences available on request).

### Immunohistochemistry

Immunohistochemistry has been performed as previously described^15^ using the following antibodies: CLIC5 (Ab75948, Abcam, Cambridge, UK), Caspase 3 (#9664 Cell Signalling Technology, London, UK), pH2AX (#9718, Cell Signalling Technology, London, UK).

### In situ hybridization

In situ hybridization was performed as described previously^48^ using Exiqon probes (Qiagen) for MIR1307 and negative control (scrambled). Color-relative staining intensities were evaluated by (1) semiquantitative evaluation considering a three-tier scale according to the intensity of the reaction (0 = negative; 1+ = faint; 2+ = moderate staining; 3+ strong and diffuse staining) and (2) ImageJ software evaluation.

### In vivo experiments

MiaPaCa-2 xenograft tumours were established subcutaneously in 6−7 week old female NOD.*Cg-Prkdc*^*scid*^*Il2rg*^*tm1Wjl*^/SzJ (NSG) mice (bred at the Institute of Cancer Research (ICR) London). Animals were housed in specific pathogen-free rooms in autoclaved, aseptic microisolator cages with a maximum of five animals per cage. A total of 8 × 10^6^ cells (WT miR-1307 and KO miR-1307) in 80% Matrigel (BD Biosciences, UK) and 20% serum-free media (Life Technologies, Paisley, UK) were injected in a single flank (Supplementary Fig. 5). Three weeks post-inoculation, mice (*n* = 16) were randomly grouped for initiation of treatment with weekly intraperitoneal (i.p.) administration of oxaliplatin (3 mg/kg), fluorouracil (25 mg/kg), and irinotecan (25 mg/kg) or vehicle (saline) alone for three weeks. Tumour volumes and mouse body weights were determined at regular intervals. Tumours were removed, weighed, and extracted for paraffin embedding. The study was performed in accordance with the UK Home Office regulations under the Animals Scientific Procedures Act 1986 and in accordance with UK National Cancer Research Institute guidelines and the NCRI guidelines^49,50^. The maximal tumour size/burden allowed (17 mm) was never exceeded.

### Reporting summary

Further information on research design is available in the Nature Research Reporting Summary linked to this article.

## Supplementary information

Supplementary Information

Description of Additional Supplementary Files

Supplementary Dataset 1

Reporting Summary

## Data Availability

All of the data is available within the Article or Supplementary Information. Source data are provided with this paper.

## Source data

Source Data
