# Supplementary material for: Modulation of pancreatic cancer cell sensitivity to FOLFIRINOX through microRNA-mediated regulation of DNA damage
Source: Nat Commun. 2021 Nov 18;12:6738. doi: 10.1038/s41467-021-27099-6 (PMC8602334; doi:10.1038/s41467-021-27099-6)
Supplement: Supplementary file 1 — Supplementary Information [file 41467_2021_27099_MOESM1_ESM.pdf]

SUPPLEMENTARTY FILE TO

Modulation of pancreatic cancer cell sensitivity to FOLFIRINOX through microRNA-mediated regulation of response to DNA damage

Short title: MIR1307 as biomarker of response to FOLFIRINOX

Pietro Carotenuto[1,2,*], Francesco Amato[3*], Andrea Lampis[4,*], Colin Rae[3], Somaieh Hedayat[4], Maria C Previdi[1], Domenico Zito[4], Maya Raj[1], Vincenza Guzzardo[5], Francesco Sclafani[6], Andrea Lanese[6], Claudia Parisi[6], Caterina Vicentini[7], Ian Said-Huntingford[1], Jens C Hahne[4], Albert Hallsworth[1], Vladimir Kirkin[1], Kate Young[6], Ruwaida Begum[6], Andrew Wotherspoon[6], Kyriakos Kouvelakis[6], Sergio Xavier Azevedo[6], Vasiliki Michalarea[6], Rosie Upstill-Goddard[3], Sheela Rao[6], David Watkins[6], Naureen Starling[6], Anguraj Sadanandam[4], David K Chang[3,8], Andrew V Biankin[3,8,9], Nigel B Jamieson[3,8], Aldo Scarpa[7], David Cunningham[6], Ian Chau[6], Paul Workman[1], Matteo Fassan[5,10], Nicola Valeri[4,6], Chiara Braconi[1,3,6,11 §]
* These authors have equally contributed to the manuscript.
[1] Division of Cancer Therapeutics, The Institute of Cancer Research, London, UK
[2] TIGEM – Telethon Institute of Genetics and Medicine, Naples, IT
[3] Institute of Cancer Sciences, University of Glasgow, Glasgow, UK
[4] Division of Molecular Pathology, The Institute of Cancer Research, London, UK
[5] Department of Medicine, University of Padua, Padua, IT
[6] The Royal Marsden NHS Trust, London and Surrey, UK
[7] ARC-Net Research Centre and Department of Diagnostics and Public Health, Section of Pathology, University of Verona, Verona, Italy
[8] West of Scotland Pancreatic Unit, Glasgow Royal Infirmary, Glasgow UK
[9] South Western Sydney Clinical School, Faculty of Medicine, University of NSW, AUS
[10] Veneto Institute of Oncology (IOV-IRCCS), Padua, IT
[11] Beatson West of Scotland Cancer Centre, Glasgow, UK

§ Contact Information: Dr Chiara Braconi, Institute of Cancer Sciences, University of Glasgow, Wolfson Wohl Cancer Research Centre, Switchback Road, Glasgow, UK G61 1QH. Phone: 0044 (0)141 330 3278. Email: chiara.braconi@glasgow.ac.uk

Supplementary Figure 1. ***Work up and quality controls of the high-throughput screening (HTS) with a library of MIR-inhibitors*****. a** Capan 1 cells were treated with scalar doses of the indicated drugs and cell viability assessed by CellTiter-Blue assay. Growth Inhibitory (GI)50 dose was calculated by using the Prism software. Bars indicate mean and SD of 6 replicates. **b** Combination of Fluorouracil, Oxaliplatin and Irinotecan (FOI) at different concentrations was tested in Capan 1 cells transfected or not with negative or positive controls. Bars represent mean and SD of seven replicates. Values from 2-sided ttest are reported. **c** FOI *b* (highest concentrations) was validated to reduce cell viability in transfected cells by <50% in MiaPaca2 cells. Bars represent mean and SD of seven replicates, normalized to DMSO. Values from 2-sided ttest are reported. **d** The MIR-inhibitors library was distributed in 5 x 384-well plates with a number of controls for plate: lack of cells, lack of transfecting reagent, lack of inhibitors (mock), two scrambled negative controls, and positive control (siTOX). Three replicates for each cell line were performed. Mean values across all plates for all three replicates for Capan 1 cells are presented. Values from 2-sided ttest are reported. **e** Data from HTS in FOI-treated Capan 1 cells are presented. Each square indicates logarithmic value of the mean of cell viability normalized to averaged negative controls (N=3), with color code indicating the degree of change in cell viability. MIR inhibitors which significantly (p<0.001) enhanced chemosensitivity by >30% in Capan 1 cells are shown. **f** Distribution of HTS controls in MiaPaca-2 cells. Values from 2-sided ttest are reported. **g** Coloured map of significant hits (see above) from HTS data for FOI-treated MiaPaca2 cells. Each row represents a replicate.

Supplementary Figure 2. ***MIR1307 is over-expressed in a subgroup of human PDAC tissues.*** **a** MIR expression was assessed in human tissues of the Panther cohort by Taqman assay. Bars represent mean and standard deviation of two technical replicates per sample. Actual values in the tumour tissue [upper panels] and logarithmic values of the ratio between the expression in the tumour tissue (TT) and the adjacent tissues (AT) [lower panels] are shown. **b** Representative pictures of H&E of the Padova cohort are reported. Top panel indicate PDAC tissues from 6 cases, while lower panel shows matched non neoplastic tissue for each case. Magnification bar indicates 100μm.

Supplementary Figure 3. ***Generation of MIR1307KO MiPaca2 cell lines*****. a** Schematic representation of the protocol followed to generate CRISPR-MIR1307 KO cell lines. **b** MiaPaca2 cells were transfected with a pair of guide RNA and a GFP construct. Forty-eight hrs later GFP positive cells were sorted. **c** Genome editing was verified as the presence of two bands. **d** Cells were enriched for the edited clones. Clone 5 was selected and named MIR1307KO. Sequencing of clone 5 is shown. **e** PCR data confirm presence of a single band. **f** TaqMan assay confirmed absence of MIR1307 expression in clone 5. Bars indicate mean and SD of 3 replicates. **g** MIR1307KO cells were treated with scalar concentrations of FOI chemotherapy. Dots represent mean and bars SD of 6 replicates. **h** WT (grey bars) and MIR1307KO MiaPaca2 (green bars) cells were treated with serial concentrations of FOI for 48 hrs. Bars represent mean and SD of 6 replicates. Values from 2-sided ttest are reported.

Supplementary Figure 4. ***Schematic representation of the irCLEAR-CLIP.*** We have developed a new protocol which involves fluorescence-based rather than radioactive-based detection of chimeras.

Supplementary Figure 5. ***Expression of CLIC5 in PDAC tissues.*** **a** irCLEAR-CLIP data are represented for WT and MIR1307KO MiaPaca2 cells after exposure to FOI treatment. The links inside the circos plots represent interaction between different RNAs within the indicated chromosomes. **b** Schematic representation of the binding site for MIR1307 within CLIC5 CDS as identified by the CLEAR-CLIP data. **c** CLIC5 protein expression was assessed by immunohistochemistry in the cases of the PANTHER study for which MIR1307 expression

(Taqman) was available. Each dot corresponds to one case with the dotted line representing the level of correlation. In the bottom panel, representative pictures of different levels of H-index for CLIC5 are shown. **d** mRNA data from the ICGC cohort were analyzed to assess the association between CLIC5 expression and the PDAC subtypes. Differentially expressed genes reported in Bailey et al[50] are shown with CLIC5 highlighted. The vertical lines correspond to logFC of 1 and -1 and the horizontal line shows p = 0.05. CLIC5 was overexpressed in classical subtype (fold change 1.37; p:0.02).

Supplementary Figure 6. *Schematic representation of in vivo experiments*. a WT or MIR1307KO MiaPaca2 cells were injected subcutaneously in the flank of NSG mice (N=16 each) and monitored for growth by caliper. At day 21 mice were randomized to be treated with a weekly intraperitoneal (i.p) vehicle (saline alone) or combination of oxaliplatin (3 mg/kg), fluorouracil (25 mg/kg) and irinotecan (25 mg/kg) for 3 weeks before being sacrificed. **b** Excide tumours from randomly selected mice were stained for MIR1307. **c** As the effect of MIR1307 was specific for FOI chemotherapy, excide tumours from FOI-treated mice were stained for CLIC5. Representative pictures are shown (left) along with quantification (right). Magnification bar indicates 100μm. MIR1307KO tumours had increased expression of CLIC5 protein.

Supplementary Table legends

Supplementary Table 1. Data from HTS. Numbers represent cell viability, expressed as mean fold change (FC) relative to NEG CTRL (N=3).

Supplementary Table 2. Clinical pathological characteristics of the PDAC cases used for the ISH analyses.

Supplementary Table 3. Data from the irCLEAR-CLIP. Hits recorded only in the WT samples.

Supplementary Table 4. Statistical analyses for the in vivo experiments. Time is presented as days since the treatment was started. Two-sided ttest was used.

Supplementary Table 5. Clinical pathological characteristics of the PDAC patients used for the analysis of circulating MIR1307.

Suppl Figure 1

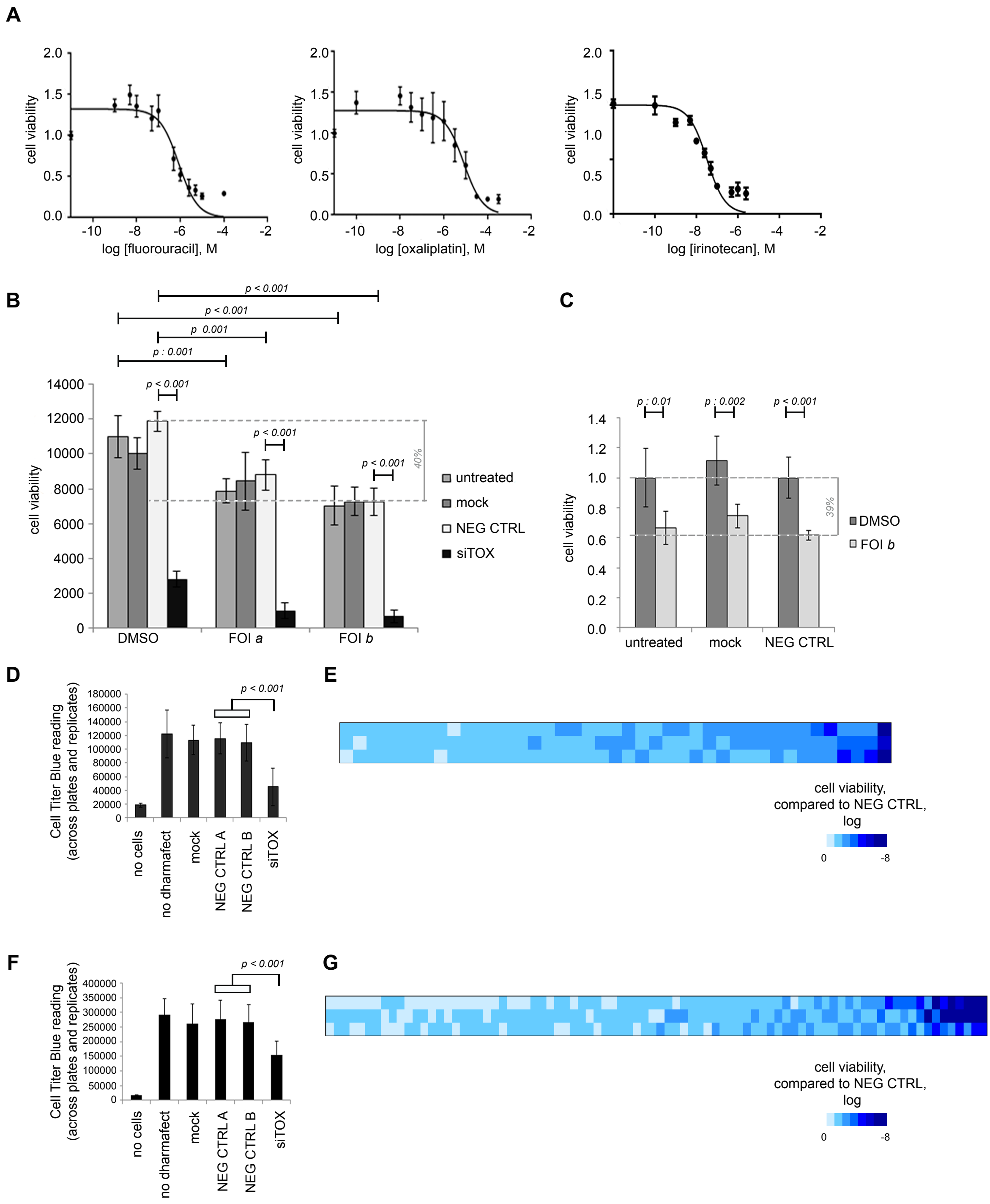

Suppl Figure 2

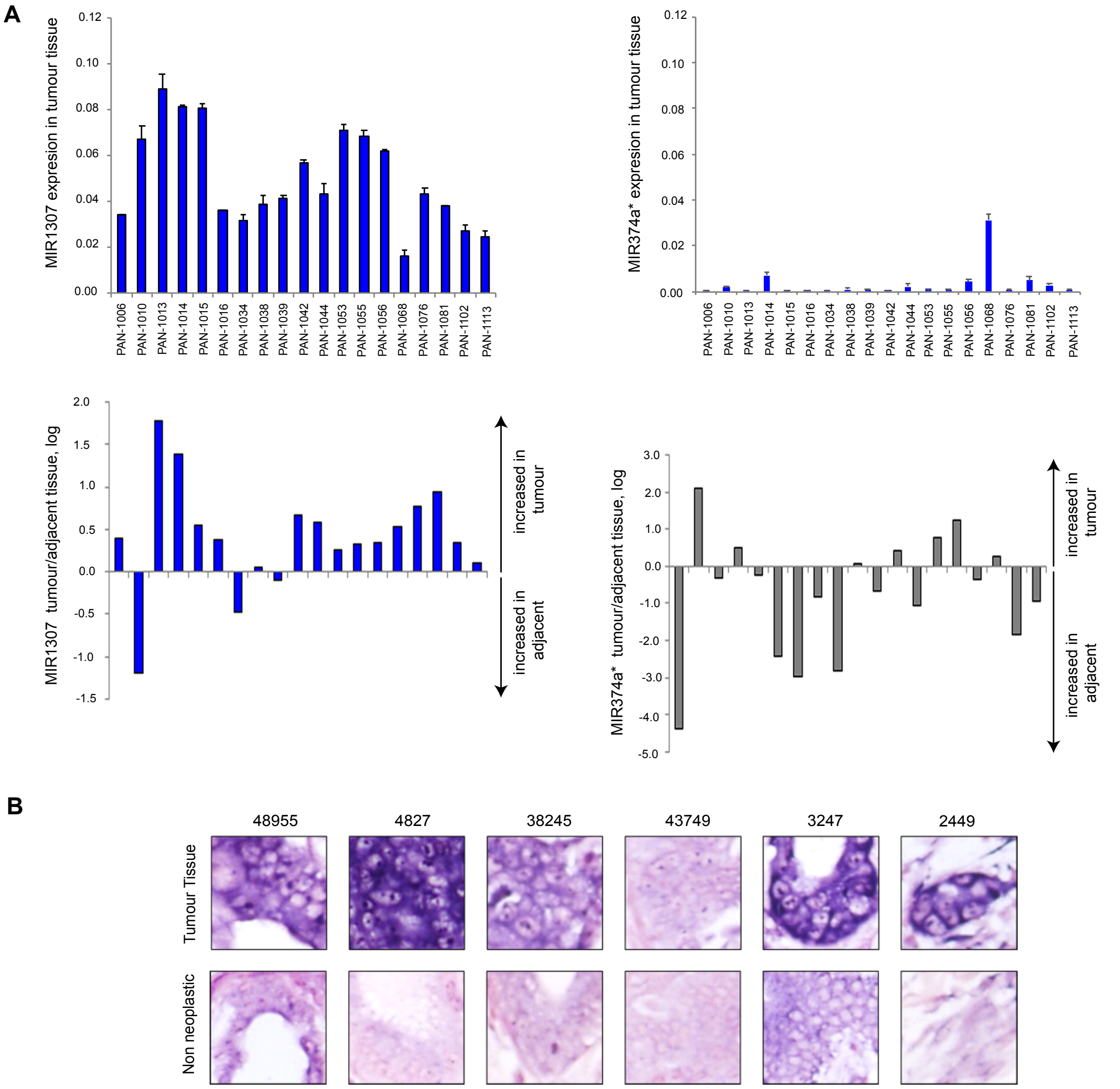

Suppl Figure 3

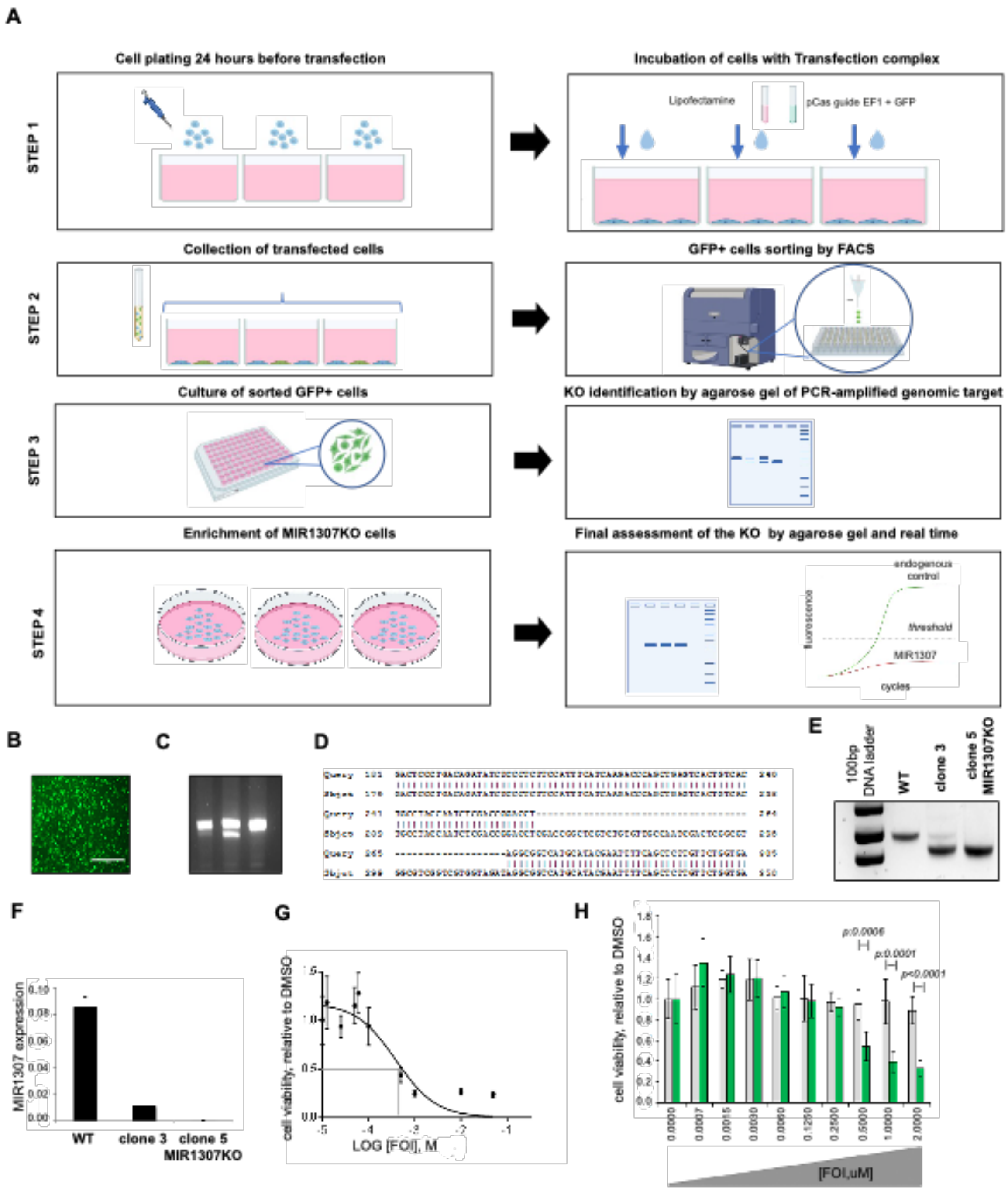

Suppl Figure 4

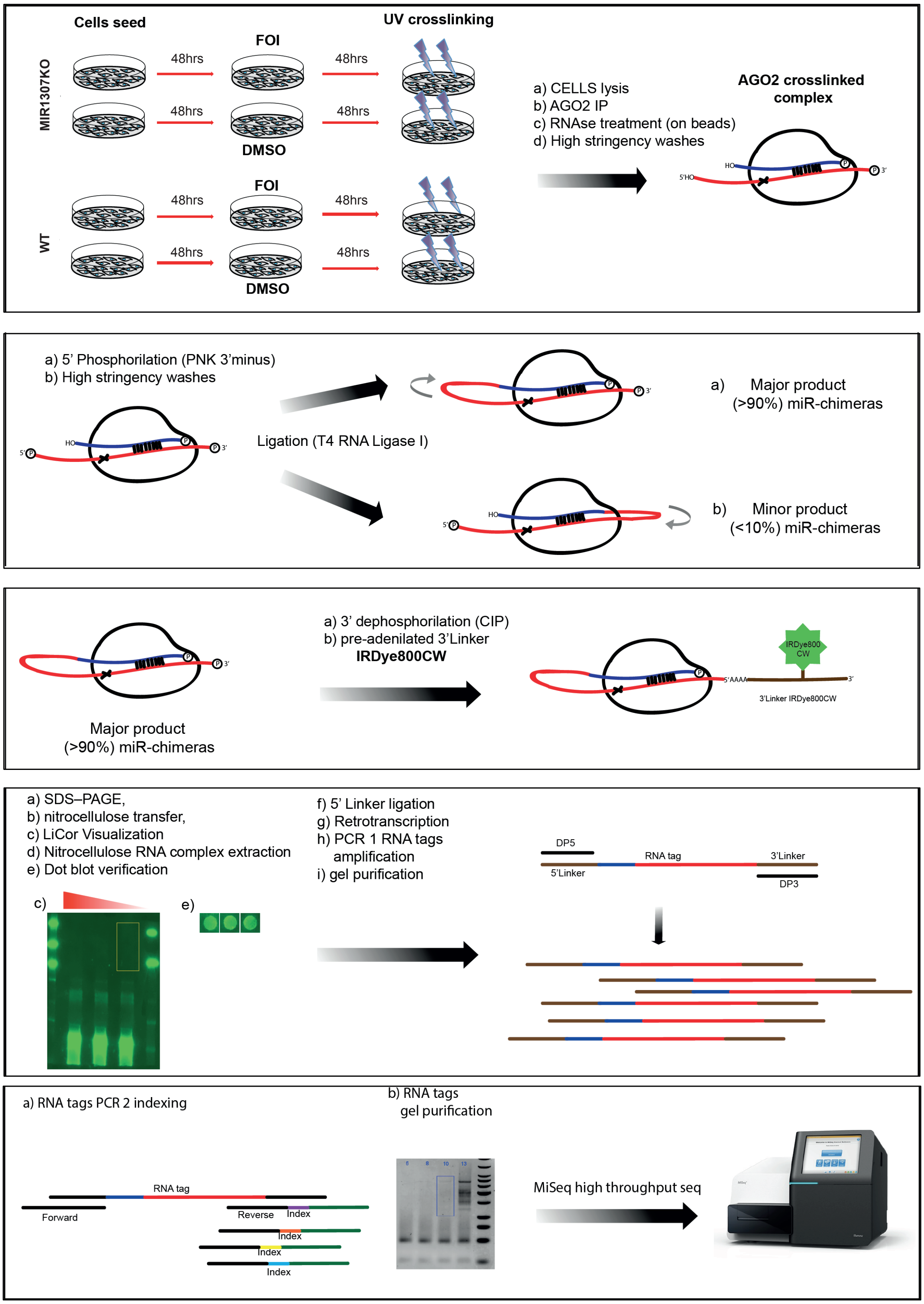

Suppl Figure 5

A

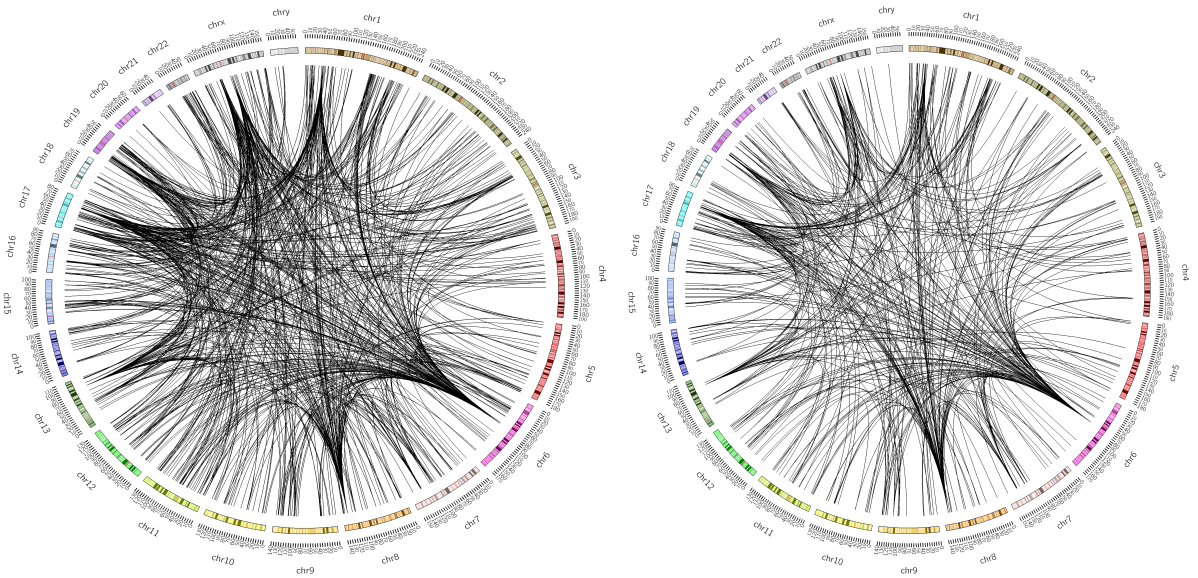

B

```
Target: CLIC5
MiRNA: hsa-miR-1307-3p
mfe: -17.7 kcal/mol
target 5' G G   A  G  AUCUCA   GAA    U 3'
           A GAC GG GC       AUG   GGGU
           U CUG CU CG       UGC   CUCA
miRNA  3' G G   G  G  G        GG       5'
```

C

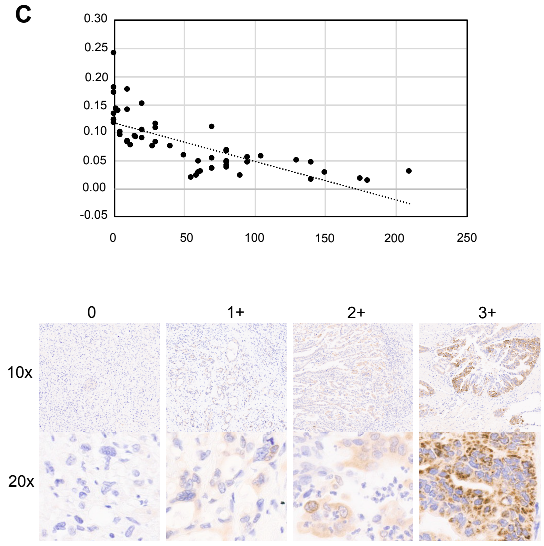

D

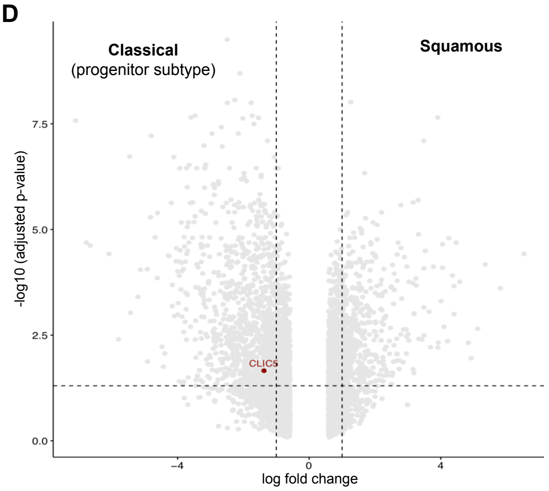

Suppl Figure 6

A

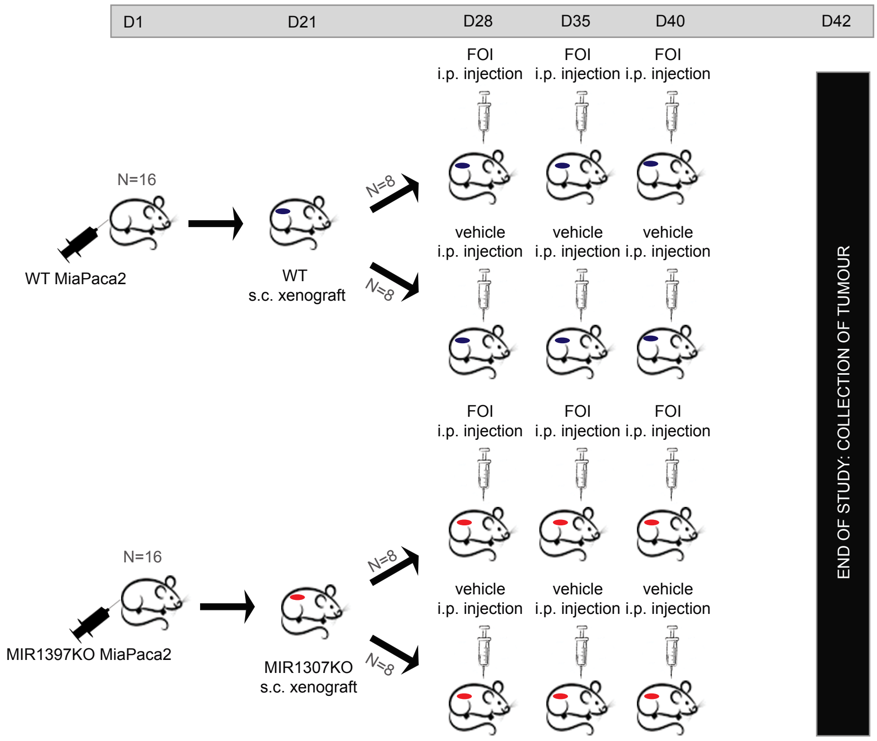

B

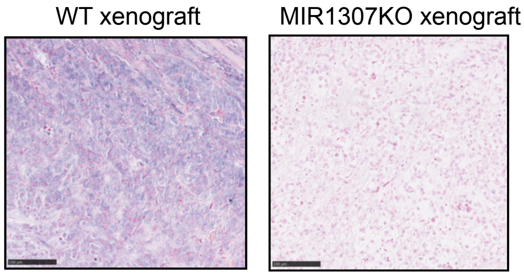

C

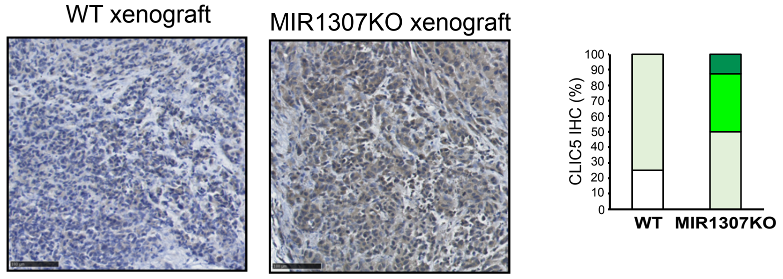

Supplementary gating strategies to Supplementary Figure 3

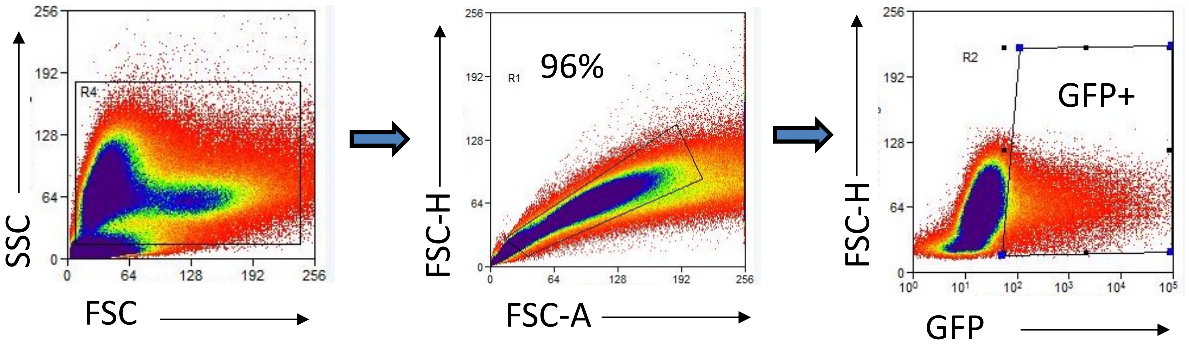

Gating strategies (referred to Suppl Figure 3). Supplementary Gating strategy for flow cytometry experiments assessing CRISPR-CAS9 GFP+ cells, previously enriched as per the methods, were gated by SSC-A vs. Single cells were chosen for analysis after doublet discrimination by detection of disproportions between cell size (FSC-A) vs. cell signal (FSC-H). Then the GFP^{+} population was plotted vs. FSC-H. GFP-positive cells can be detected outside of the negative population of cells measured with a 488-530 nm laser.

Uncropped PCR gel to Supplementary Figure 3C

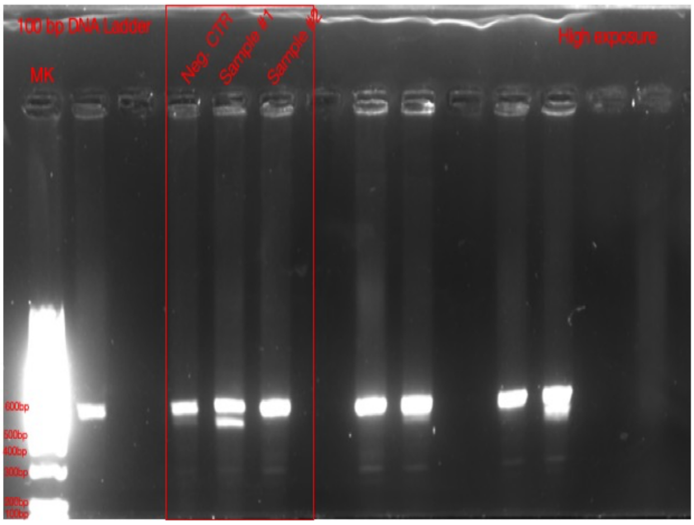

Supplementary Table 1. Data from HTS. Numbers represent cell viability, expressed as mean fold change (FC) relative to NEG CTRL (N=3).

	Capan1		MiaPaCa2	
	FC (mean)	p value	FC (mean)	p value
MIR1247	1.22	0.512	0.73	0.140
MIR374a*	0.34	0.003	0.76	0.119
MIR1307	0.35	<0.001	0.62	0.003
MIR944	0.43	0.001	0.68	0.001

Supplementary Table 2. Clinical pathological characteristics of the PDAC cases used for the ISH analyses

	number	%
Gender		
female	4	66
male	2	34
Age		
median	70	
Grade		
G3	6	100
Pathological stage		
II A	1	17
II B	5	83
Tumour MIR1307 score		
0 / 1+	1	17
2+ / 3+	5	83

Supplementary Table 3. Data from the irCLEAR-CLIP. Hits recorded only in the WT samples.

microRNA	Mapping Position	V5	V6	Gene Lenght	gene ID	UTR5 Start	UTR5 Stop	CDS Start	CDS Stop	UTR3 Start	UTR3 Stop	Binding Location
hsa-miR-1307-3p	*322*	*255*	*22M4S*	*6023*	*CLIC5*	*1*	*153*	*154*	*1386*	*1387*	*6023*	*CDS*
hsa-miR-1307-3p	*564*	*0*	*4S20M3S*	*10757*	*ITGA1*	*1*	*458*	*459*	*3998*	*3999*	*10757*	*CDS*
hsa-miR-1307-3p	*1117*	*3*	*9S18M*	*1315*	*RAB8A*	*1*	*5*	*6*	*629*	*630*	*1315*	*UTR3*
hsa-miR-1307-3p	*895*	*255*	*1S25M*	*5353*	*STC2*	*1*	*1310*	*1311*	*2219*	*2220*	*5353*	*UTR5*
hsa-miR-1307-3p	*1217*	*1*	*24M*	*1926*	*STX11*	*1*	*183*	*184*	*1047*	*1048*	*1926*	*UTR3*

Supplementary Table 4. Statistical analyses for the in vivo experiments. Time is presented as days since the treatment was started.

	DAY 7	DAY 14	DAY 19	DAY 21
**WT FOI** vs **WT vehicle**	0.092	0.001	0.009	0.032
**MIR1307KO veichle** vs **MIR1307KO FOI**	0.081	0.067	0.010	0.016
**MIR1307KO vehicle** vs **WT vehicle**	0.794	0.524	0.029	0.040
**FOI MIR1307KO** vs **FOI WT**	0.886	0.838	0.008	0.011

Supplementary Table 5. Clinical pathological characteristics of the PDAC patients enrolled for the analysis of circulating MIR1307

	number	%
Gender		
female	6	33
male	12	67
Age		
median	67	
Stage of disease		
locally advanced	8	44
metastatic	10	56
Performance status		
ECOG 0	15	83
ECOG 1	3	17
