# Supplementary material for: Modulation of pancreatic cancer cell sensitivity to FOLFIRINOX through microRNA-mediated regulation of DNA damage
Source: Nat Commun. 2021 Nov 18;12:6738. doi: 10.1038/s41467-021-27099-6 (PMC8602334; doi:10.1038/s41467-021-27099-6)
Supplement: Supplementary file 2 — Description of Additional Supplementary Files [file 41467_2021_27099_MOESM2_ESM.docx]

**Supplementary Dataset 1. HTS data for selected miRNA inhibitors**. Numbers represent cell viability, expressed as mean fold change (FC) relative to NEG CTRL (N=3). Values from 2-sided ttest are reported.
