# Supplementary material for: Modulation of pancreatic cancer cell sensitivity to FOLFIRINOX through microRNA-mediated regulation of DNA damage
Source: Nat Commun. 2021 Nov 18;12:6738. doi: 10.1038/s41467-021-27099-6 (PMC8602334; doi:10.1038/s41467-021-27099-6)
Supplement: Supplementary file 4 — Reporting Summary [file 41467_2021_27099_MOESM4_ESM.pdf]

nature portfolio

Corresponding author(s): NCOMMS-20-37735A BRACONI, Chiara

Last updated by author(s): Oct 8, 2021

Reporting Summary

Nature Portfolio wishes to improve the reproducibility of the work that we publish. This form provides structure for consistency and transparency in reporting. For further information on Nature Portfolio policies, see our Editorial Policies and the Editorial Policy Checklist.

Statistics

For all statistical analyses, confirm that the following items are present in the figure legend, table legend, main text, or Methods section.

n/a	Confirmed	
☐	☒	The exact sample size (n) for each experimental group/condition, given as a discrete number and unit of measurement
☐	☒	A statement on whether measurements were taken from distinct samples or whether the same sample was measured repeatedly
☐	☒	The statistical test(s) used AND whether they are one- or two-sided<br>*Only common tests should be described solely by name; describe more complex techniques in the Methods section.*
☐	☒	A description of all covariates tested
☐	☒	A description of any assumptions or corrections, such as tests of normality and adjustment for multiple comparisons
☐	☒	A full description of the statistical parameters including central tendency (e.g. means) or other basic estimates (e.g. regression coefficient) AND variation (e.g. standard deviation) or associated estimates of uncertainty (e.g. confidence intervals)
☐	☒	For null hypothesis testing, the test statistic (e.g. F, t, r) with confidence intervals, effect sizes, degrees of freedom and P value noted<br>*Give P values as exact values whenever suitable.*
☒	☐	For Bayesian analysis, information on the choice of priors and Markov chain Monte Carlo settings
☒	☐	For hierarchical and complex designs, identification of the appropriate level for tests and full reporting of outcomes
☒	☐	Estimates of effect sizes (e.g. Cohen's d, Pearson's r), indicating how they were calculated

Our web collection on statistics for biologists contains articles on many of the points above.

Software and code

Policy information about availability of computer code

Data collection	BD FACSDiva v9.0 Software to collect data from BD FACSAria II (BD Biosciences, San Jose, CA, USA)
Data analysis	Prism Software (Graphpad, La Jolla, USA), v 6.0<br>ImageJ software, version bundled with Java 1.8.0_172<br>BD FACSDiva v9.0 Software

For manuscripts utilizing custom algorithms or software that are central to the research but not yet described in published literature, software must be made available to editors and reviewers. We strongly encourage code deposition in a community repository (e.g. GitHub). See the Nature Portfolio guidelines for submitting code & software for further information.

Data

Policy information about availability of data

All manuscripts must include a data availability statement. This statement should provide the following information, where applicable:
- Accession codes, unique identifiers, or web links for publicly available datasets
- A description of any restrictions on data availability
- For clinical datasets or third party data, please ensure that the statement adheres to our policy

All data are available in the figures and supplementarty files.

Field-specific reporting

Please select the one below that is the best fit for your research. If you are not sure, read the appropriate sections before making your selection.

☒ Life sciences ☐ Behavioural & social sciences ☐ Ecological, evolutionary & environmental sciences

For a reference copy of the document with all sections, see nature.com/documents/nr-reporting-summary-flat.pdf

Life sciences study design

All studies must disclose on these points even when the disclosure is negative.

Sample size	This is not a clinical trial. Sample size was chosen on the basis of the experiments. Biological replicates from independent experiments are reported in the text and figures. When technical replicates were available are presented and specified. Patients' samples were from retrospective cohorts and size was dependent on the consecutive cases observed in the conditions specified in the text. Descriptive statistical analysis is described for these cohorts and as such no sample size prediction was necessary.
Data exclusions	Data were not excluded
Replication	Experiments were reproduced in independent biological experiments, as provided in the legend of each figure. Two cell lines were used for engineered experiments. Clinical relevance was validated in >3 patients' cohorts.
Randomization	not applicable because there are no prospective clinical trials
Blinding	not applicable because there are no prospective clinical trials

Reporting for specific materials, systems and methods

We require information from authors about some types of materials, experimental systems and methods used in many studies. Here, indicate whether each material, system or method listed is relevant to your study. If you are not sure if a list item applies to your research, read the appropriate section before selecting a response.

Materials & experimental systems

n/a	Involved in the study	
☐	☒	Antibodies
☐	☒	Eukaryotic cell lines
☒	☐	Palaeontology and archaeology
☐	☒	Animals and other organisms
☐	☒	Human research participants
☒	☐	Clinical data
☒	☐	Dual use research of concern

Methods

n/a	Involved in the study	
☒	☐	ChIP-seq
☐	☒	Flow cytometry
☒	☐	MRI-based neuroimaging

Antibodies

Antibodies used	The following primary antibodies were used for western blot: rabbit anti-human PARP (Cell Signaling Technology, London, UK; #9542; dil. 1:1000), rabbit anti-human PARP cleaved (Cell Signaling Technology, London, UK; #5625; dil. 1:1000), rabbit anti-human Caspase 3 (Cell Signaling Technology, London, UK; #9662; 1:1000), rabbit anti-human Caspase 3 cleaved (Cell Signaling Technology, London, UK; #9664; 1:1000), rabbit anti-human Phospho-Histone H2A.X (Ser139) (Cell Signaling Technology, London, UK; #9718; 1:1000), as well as CLIC5 (Sigma-Aldrich, Gillingham, UK; #AV35262, 1:1000) and Beta-Actin (MP Biomedicals, Loughborough UK; #8691002, Clone C4; 1:1000).<br>The following antibodies were used for IF and IHC staining: mouse anti-human pATM (Biolegend, San Diego USA; #Clone 10H11.E12), mouse anti-human H2Ax (ser139) Alexa Fluor 647 (Biolegend, San Diego USA; #Clone2F3; 1:200), rabbit anti-mouse Alexa Fluor 568 (Abcam, Cambrigde UK; #ab206901; 1:200), rabbit anti-human CLIC5 (Abcam, Cambridge, UK, #Ab75948; 1:500), rabbit anti-human Caspase 3 (Cell Signaling Technology, London, UK; ##9664, dil 1:1000), rabbit anti-human pH2AX (Cell Signaling Technology, London, UK; ##9718, dil 1:1000).
Validation	For the immunohistochemistry of CLIC5 (Sigma-Aldrich, Gillingham, UK; #AV35262, 1:1000) we validated the antibody in intestine tissue. Positive controls have been introduced for the assessment of apoptosis. Further detailed antibody validation information is available from the manufacturers.

Eukaryotic cell lines

Policy information about cell lines

Cell line source(s)	The following PDAC cell lines were purchased from the American Type Culture Collection (ATCC):<br>Capan-1 (ATCC HTB-79)<br>MIA PaCa-2 (ATCC CRM-CRL-1420)<br>PANC-1 (ATCC CRL-1469)<br>AsPC-1 (ATCC CRL-1682)<br>BxPC-3 (ATCC CRL-1687)<br>SU.86.86 (ATCC CRL-1837)
Authentication	Cells were authenticated through Short Tandem Repeat (STR) analysis.
Mycoplasma contamination	Cells were regularly tested negative for Mycoplasma.
Commonly misidentified lines<br>(See ICLAC register)	No commonly misidentified lines were used

Animals and other organisms

Policy information about studies involving animals; ARRIVE guidelines recommended for reporting animal research

Laboratory animals	6-7 week old female NOD.Cg-PrkdcscidIl2rgtm1Wjl/SzJ (NSG) mice (bred at the Institute of Cancer Research (ICR) London). Animals were housed in specific pathogen-free rooms in autoclaved, aseptic microisolator cages with a maximum of five animals per cage.
Wild animals	not used
Field-collected samples	not used
Ethics oversight	The study was performed in accordance with the UK Home Office regulations under the Animals Scientific Procedures Act 1986 and in accordance with UK National Cancer Research Institute guidelines and the NCRI guidelines

Note that full information on the approval of the study protocol must also be provided in the manuscript.

Human research participants

Policy information about studies involving human research participants

Population characteristics	Retrospective cohorts:<br>1- Royal Marsden retrospective cohort of human resected PDAC (Panther) - FFPE tissues<br>2- University of padua retrospective cohort of human resected PDAC -FFPE tissues<br>3- Royal Marsden retrospective cohort of patients undergoing Folfirinox chemotherapy - plasma<br><br>Demographic characteristics are provided in Suppl Table 3 and 6.
Recruitment	retrospective cohorts.
Ethics oversight	The human PDAC tissues were collected under approval of the Ethical Committee for Clinical Research at the Royal Marsden NHS Trust (Panther study: CCR 4192). A second cohort from the University Hospital of Padua (ethics #0010416) was used for the in-situ hybridization. For circulating miR assessment patients with unresectable pancreatic carcinoma undergoing FOLFIRINOX chemotherapy were enrolled under the research protocol CCR3085 (SSGCC) that has received approval from the Research Ethics Committee, London. All patients provided informed consent. None of the patients received participation compensation.

Note that full information on the approval of the study protocol must also be provided in the manuscript.

Flow Cytometry

Plots

Confirm that:

- [x] The axis labels state the marker and fluorochrome used (e.g. CD4-FITC).
- [x] The axis scales are clearly visible. Include numbers along axes only for bottom left plot of group (a 'group' is an analysis of identical markers).
- [x] All plots are contour plots with outliers or pseudocolor plots.
- [x] A numerical value for number of cells or percentage (with statistics) is provided.

Methodology

Sample preparation	• Samples are preferred in sterilized PBS with 2% FBS/PBS.<br>• For optimum sorting and saving your precious samples, a diluted aliquot (300-400ul total) of your cells-to-be sorted should be placed in a separate control tubes in order to set up the instrument.<br>Controls:<br>Negative unstained controls (0.3ml minimum volume)
Instrument	BD FACSAria II (BD Biosciences, San Jose, CA, USA)
Software	BD FACSDiva v9.0 Software to collect data from BD FACSAria II (BD Biosciences, San Jose, CA, USA)
Cell population abundance	Abundance of cells was higher than 95% and Purity verified by used controls and by analysing cells with different methods.
Gating strategy	Compensation controls for each fluorochrome (0.3ml minimum volume) if necessary.<br>Acquisition Samples Volume:<br>Cells for sorting should be in 0.5ml minimum volume if you have less than 1x106 cells.<br>Usually 5-10 million per ml PBS.<br>Data acquisition/Record runs typically only acquire 10,000~30,000 gated events.<br><br>Gating Strategy to sort CRISPR-CAS9/GFP positive live cells.<br>Gating strategy for flow cytometry experiments assessing CRISPR-CAS9 GFP+ cells, previously enriched as per the methods, were gated by SSC-A vs. Single cells were chosen for analysis after doublet discrimination by detection of disproportions between cell size (FSC-A) vs. cell signal (FSC-H). Then the GFP^+ population was plotted vs. FSC-H. GFP-positive cells can be detected outside of the negative population of cells measured with a 488-530 nm laser.<br><br>A figure for gating strategies has been added to the source data

☒ Tick this box to confirm that a figure exemplifying the gating strategy is provided in the Supplementary Information.
