# Supplementary material for: Modulation of pancreatic cancer cell sensitivity to FOLFIRINOX through microRNA-mediated regulation of DNA damage
Source: Nat Commun. 2021 Nov 18;12:6738. doi: 10.1038/s41467-021-27099-6 (PMC8602334; doi:10.1038/s41467-021-27099-6)

**SOURCE DATA TO**

Figure 6c

(The membrane had been cut before proceeding with the staining in order to proceed with the probing for vinculin and CLIC-5)


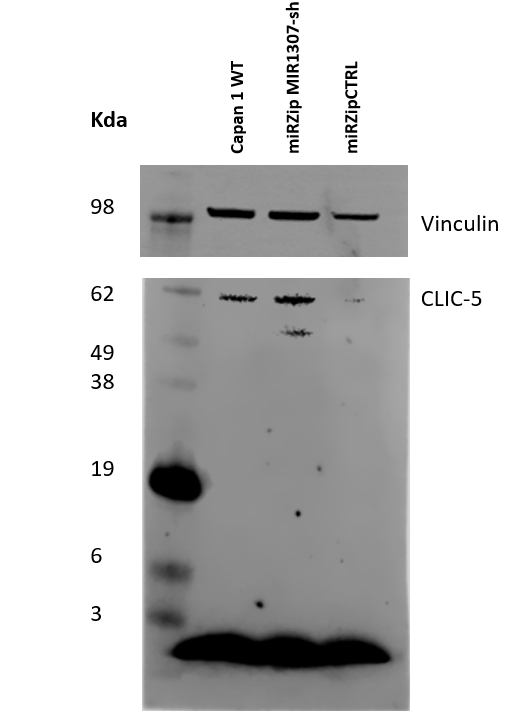

Supplement: Supplementary file 5 — Source Data [file 41467_2021_27099_MOESM5_ESM.zip › Source data /6. Source data Figure 6.docx]
